# Homology-based repair induced by CRISPR-Cas nucleases in mammalian embryo genome editing

**DOI:** 10.1007/s13238-021-00838-7

**Published:** 2021-05-04

**Authors:** Xiya Zhang, Tao Li, Jianping Ou, Junjiu Huang, Puping Liang

**Affiliations:** 1 Center for Reproductive Medicine, the Third Affiliated Hospital of Sun Yat-sen University, Sun Yat-sen University, Guangzhou, 510630 China; 2 MOE Key Laboratory of Gene Function and Regulation, State Key Laboratory of Biocontrol, School of Life Sciences, Sun Yat-sen University, Guangzhou, 510275 China; 3 Key Laboratory of Reproductive Medicine of Guangdong Province, the First Affiliated Hospital and School of Life Sciences, Sun Yat-sen University, Guangzhou, 510275 China

**Keywords:** homology-based repair (HBR), genome editing, disease modeling, embryo, precision medicine

## Abstract

Recent advances in genome editing, especially CRISPR-Cas nucleases, have revolutionized both laboratory research and clinical therapeutics. CRISPR-Cas nucleases, together with the DNA damage repair pathway in cells, enable both genetic diversification by classical non-homologous end joining (c-NHEJ) and precise genome modification by homology-based repair (HBR). Genome editing in zygotes is a convenient way to edit the germline, paving the way for animal disease model generation, as well as human embryo genome editing therapy for some life-threatening and incurable diseases. HBR efficiency is highly dependent on the DNA donor that is utilized as a repair template. Here, we review recent progress in improving CRISPR-Cas nuclease-induced HBR in mammalian embryos by designing a suitable DNA donor. Moreover, we want to provide a guide for producing animal disease models and correcting genetic mutations through CRISPR-Cas nuclease-induced HBR in mammalian embryos. Finally, we discuss recent developments in precise genome-modification technology based on the CRISPR-Cas system.

**Supplementary Information:**

The online version of this article (10.1007/s13238-021-00838-7) contains supplementary material, which is available to authorized users.

## Introduction

Genome editing, capable of rewriting DNA sequences *in situ*, holds tremendous potential in research and clinical applications. Genome editing in animal embryos provides an efficient way to generate genome-modified mammalian animals (e.g., mice, rats, rabbits, pigs, and monkeys), which holds tremendous potential in disease modeling and precision medicine (Anzalone et al., 2020; Doudna, 2020). Furthermore, genome editing in human embryos may be an option to prevent genetic disease transmission and save lives once the technical, safety, ethical, social, and legal issues associated with human embryo genome editing have been resolved (Baltimore et al., 2015; Rossant, 2018; Macintosh, 2019).

**Figure 1:**
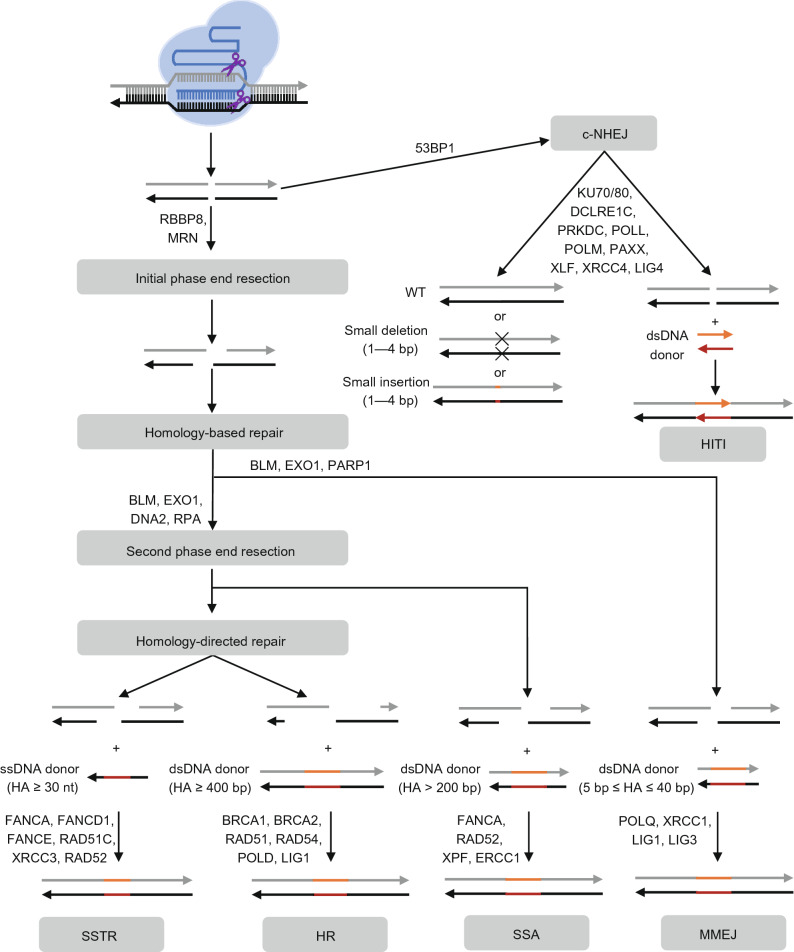
DNA repair pathways involved in CRISPR-Cas nucleases mediated genome editing in mammalian cells. CRISPR-Cas nuclease-induced DNA DSBs are repaired by either c-NHEJ or homology-based repair (HBR). The recruitment of 53BP1 inhibits end resection at the DSB site, promoting DSB repair via the c-NHEJ pathway. Without exogenous DNA donors, the two ends of the DSB are ligated together precisely or imprecisely with 1–4 bp small insertion or deletion (indel) through the c-NHEJ pathway. In the presence of a double-stranded DNA (dsDNA) donor without homology arms, the dsDNA donor can be inserted into the DSB site via the c-NHEJ pathway (HITI). In addition, the two ends of the DSB site may undergo initial phase end resection by RBBP8 (or named CtIP) and MRN, generating short 3′ overhang. Supplied with a DNA donor with homology arms, the 3′ overhang will prime DNA repair through the high-fidelity homology-based repair pathway. Supplied with a dsDNA donor with short homology arms (5–40 bp), the DSB site could be repaired via the MMEJ pathway. Furthermore, the short 3′ overhang may undergo second phase end resection, generating a longer 3′ overhang. In the presence of dsDNA donor with medium homology arms (>200 bp), the DSB site could be repaired via the SSA pathway. Supplied with a dsDNA donor with long homology arms (≥400 bp), the DSB site could be repaired via the HR pathway. In the presence of ssDNA donor with short homology arms (≥30 nt), the DSB site could be repaired via the SSTR pathway. Some major proteins involved in each pathway are shown in the figure. The direction of the arrow represents the 5′ to 3′ direction. Deletion, black cross. Inserted fragment, colored line. c-NHEJ, classical nonhomologous end joining. HITI, homology-independent targeted integration. MRN, MRE11-RAD50-NBS1 complex. HA, homology arm. SSTR, single-stranded templated repair. HR, homologous recombination. SSA, single-stranded annealing. MMEJ, microhomology-mediated end joining

Since the first report of genome editing by meganuclease in the 1990s (Rouet et al., 1994; Choulika et al., 1995), zinc finger nuclease (ZFN), transcription activator-like effector nuclease (TALEN), and clustered regularly interspaced short palindromic repeat (CRISPR)-CRISPR-associated (CRISPR-Cas) nucleases have been utilized for genome editing (Urnov et al., 2010; Gaj et al., 2013). Among these four kinds of genome editing tools, CRISPR-Cas nucleases stand out as the most convenient, cost-effective, versatile, and robust tool (Anzalone et al., 2020). CRISPR-Cas nuclease (e.g., Cas9, Cas12a, Cas12b, and CasX), adapted from the adaptive immune system of bacteria and archaea, is an RNA-protein complex composed of guide RNA (gRNA) and Cas protein (Jinek et al., 2012; Cong et al., 2013; Mali et al., 2013). The gRNA-Cas complex searches for the target DNA containing both the protospacer adjacent motif (PAM) sequence and the sequence complementary to the gRNA (Sternberg et al., 2015; Jiang et al., 2016; Palermo et al., 2016, 2017; Chen et al., 2017). The guide sequence at the end of the gRNA pairs with the complementary strand (or named target strand) of the target DNA, resulting in CRISPR-Cas nuclease activation and DNA double-stranded breaks (DSBs) (Sternberg et al., 2015; Jiang et al., 2016; Palermo et al., 2016, 2017; Chen et al., 2017). DNA DSBs generated by CRISPR-Cas nuclease can be repaired by either classical non-homologous end joining (c-NHEJ) or homology-directed repair (HBR) (Fig. 1) (Cong et al., 2013; Mali et al., 2013). c-NHEJ will result in random small deletions or insertions at the target site. In addition, in the presence of an exogenous DNA donor, c-NHEJ may lead to DNA donor integration at the target site without homology arms (homology-independent targeted integration, HITI) (Fig. 1) (Suzuki et al., 2016). However, HITI tends to induce mutations at the junctions between the DNA donor and the target site (Suzuki et al., 2016). Provided with single-stranded oligodeoxynucleotides (ssODN), whose homology arm could pair with both the DNA donor and the target site, further increased the targeted integration efficiency of HITI (Yoshimi et al., 2016). HBR includes homology-directed repair (HDR), microhomology-mediated end joining (MMEJ, or alternative end-joining), and single-stranded annealing (SSA) (Bennardo et al., 2008; Ceccaldi et al., 2016; Chang et al., 2017). HDR can be further divided into single-stranded templated repair (SSTR) and homologous recombination (HR), depending on the donor DNA type (Fig. 1) (Sakuma and Yamamoto, 2017; Richardson et al., 2018; Yeh et al., 2019). HBR can utilize exogenous DNA donors with homology arms to repair DSB, resulting in the precisely edited target genes, including precise deletion, precise point mutation, tag (or reporter gene) integration, and conditional allele generation (Yang et al., 2013). To prevent re-cutting of the edited allele, the exogenous DNA donor should contain both the desired sequence change and blocking silent mutations, which will prevent the gRNA-Cas complex from cleaving the edited allele (Paquet et al., 2016; Kwart et al., 2017). It is noteworthy that MMEJ and SSA can also occur between two homologous sequences flanking the target site, deleting the intervening sequence (Shen et al., 2018; Zhang and Matlashewski, 2019). Although recently developed base editors and prime editors could realize some of the functions (e.g., precise C-to-T conversion, precise A-to-G conversion, precise C-to-G conversion, small DNA fragment deletion, and small DNA fragment insertion) of CRISPR-Cas nuclease-induced HBR, they could not be utilized to catalyze other types of base conversion (e.g., G-to-C conversion), inserting large DNA fragments, and generating a conditional allele (Komor et al., 2016; Gaudelli et al., 2017; Anzalone et al., 2019; Kurt et al., 2020; Zhao et al., 2020a). However, HBR is much less efficient than c-NHEJ in mammalian embryos, resulting in mosaicism in edited embryos. Mosaic embryos contain precisely edited cells, imperfectly edited cells, and non-edited cells. For small mammalian animals, such as mice, rats, and rabbits, homozygous edited animals could be generated by repeated breeding. However, repeated breeding is a bottleneck for large mammalian animal model generation, which requires a longer time to reach sexual maturity (e.g., pigs and monkeys). Moreover, mosaicism and unintended editing are the main technical issues that impede the clinical application of human embryo genome editing therapy (Zuccaro et al., 2020). Improving CRISPR-Cas nuclease-induced HBR efficiency will mitigate mosaicism in embryo genome editing (Zuccaro et al., 2020). Taken together, it is of great significance to develop new methodologies to improve HBR efficiency in mammalian embryos.

There have already been excellent reviews on recent advances in improving HBR repair by controlling DNA repair pathways (Liu et al., 2018a; Yeh et al., 2019). The DNA donor is a critical determinant of HBR efficiency. In this review, we restrict our discussion to the topic of improving HBR by designing suitable DNA donors for genome editing in mammalian embryos, especially in frequently investigated rodent embryos. There are several types of DNA donors, including single-stranded DNA (ssDNA), double-stranded plasmid, and linear double-stranded DNA (dsDNA). Genome editing outcomes in embryos are believed to be similar between mice and large mammalian animals. Comparative analysis of these data will provide a practical guide for embryo gene editing in large mammalian embryos (Ma et al., 2017a, 2018; Adikusuma et al., 2018; Wilde et al., 2018; Zuccaro et al., 2020).

## Homology-based repair using single-stranded DNA donor

Single-stranded DNA (ssDNA) donors include ssODN, long single-stranded DNA (lssDNA), and single-stranded adeno-associated virus (AAV) genomic DNA. ssODN, which is typically no longer than 200 nt, could be synthesized by a commercial company. lssDNA is an ssDNA longer than 200 nt and could be generated by *in vitro* transcription and reverse transcription (ivTRT) (Miura et al., 2015; Quadros et al., 2017; Codner et al., 2018; Li et al., 2019), chemical synthesis (Quadros et al., 2017), selection of ssDNA labeled with biotin (Stahl et al., 1988), and the strand-specific digestion of dsDNA (Murgha et al., 2014; Yoshimi et al., 2016). AAV is a single-stranded DNA virus with a genome of approximately 4.8 kb. AAV could be produced by transfecting HEK-293T cells with double-stranded AAV plasmid vector, Rep-Cap plasmid, and helper plasmid (Chen et al., 2020). Upon AAV infection, single-stranded AAV genomic DNA is released into the cell and works as a repair template (Yoon et al., 2018; Chen et al., 2019).

Compared with double-stranded DNA (dsDNA) donors, such as plasmids and linear dsDNA, the knock-in (KI) efficiency using single-stranded DNA (ssDNA) donor is much higher (Miura et al., 2015; Codner et al., 2018). Owing to the SSTR pathway utilized by ssDNA donors, the homology arm of ssDNA donors is much shorter than plasmid and linear dsDNA, which enables the convenient and high-throughput construction of DNA donors (Kan et al., 2017). These two features make ssDNA an ideal DNA donor.

### HBR using ssODN

ssODN could be used as a repair template to generate precise modifications, such as point mutations, small insertions, or precise deletions. To achieve high editing efficiency, the distance between the modification and the Cas nuclease cleavage site should be as small as possible (≤30 bp) (Renaud et al., 2016; Quadros et al., 2017). Generally, ssODN contains a ≥30-nt homology arm at both the 5′ and 3′ end, where a longer homology arm could increase the editing efficiency (Renaud et al., 2016). However, ssODN with a longer homology arm also displays more severe cytotoxicity (Okamoto et al., 2019).

Previous genome editing studies using ZFN and TALEN have proven that ssODN could be used as a DNA donor to generate precisely edited cells and animal models (Chen et al., 2011; Bedell et al., 2012; Shen et al., 2013). Combing Cas9 nuclease with ssODN enables the efficient insertion of a restriction enzyme recognition site in human cells (Cong et al., 2013; Ran et al., 2013). Delivering Cas9 nuclease together with ssODN has previously led to the efficient correction of *HBB* and *CYBB* mutations in human hematopoietic stem and progenitor cells (HSPCs) (DeWitt et al., 2016; De Ravin et al., 2017). It is reported phosphorothioate (PS) bound at the several terminal nucleotides of ssODN could improve targeted insertion efficiency by protecting the ssODN from degradation (Papaioannou et al., 2009), and was previously found to improve the repair efficiency of the *CYBB* mutation in HSPCs (De Ravin et al., 2017). However, the repair efficiency of *HBB* mutations was not improved by phosphorothioate (PS) modification (DeWitt et al., 2016). Thus, whether phosphorothioate (PS) modification improves the targeted insertion efficiency is likely to depend on the ssODN used. In addition to phosphorothioate (PS) modification, locked nucleic acid (LNA) modification could also enhance KI efficiency in human cells by improving the stability of ssODN (Renaud et al., 2016).

Co-injecting ssODN together with CRISPR-Cas nuclease into zygotes leads to highly efficient KI of small fragments in mouse, rat, rabbit, pig, sheep, bovine, and even human embryos (Table S1). In line with the cellular data, PS-modified ssODN also showed improved KI efficiency in mouse and rat embryos (Table S1) (Renaud et al., 2016). However, whether PS- or LNA-modified ssODN improves the KI efficiency in large mammalian animal and human embryos remains to be investigated.

One limitation of ssODN is its limited length (~200 nt), making it impossible to achieve larger DNA fragment KI. Although using multiple ssODNs with overlapping regions allows larger fragment KI in *C*. *elegans*, overlapping ssODNs generated incomplete KI in rats (Paix et al., 2016; Remy et al., 2017). Whether ssODNs with overlapping regions could result in larger DNA fragment KI in the emfbryos of large mammalian animals and humans remains unclear.

**Figure 2:**
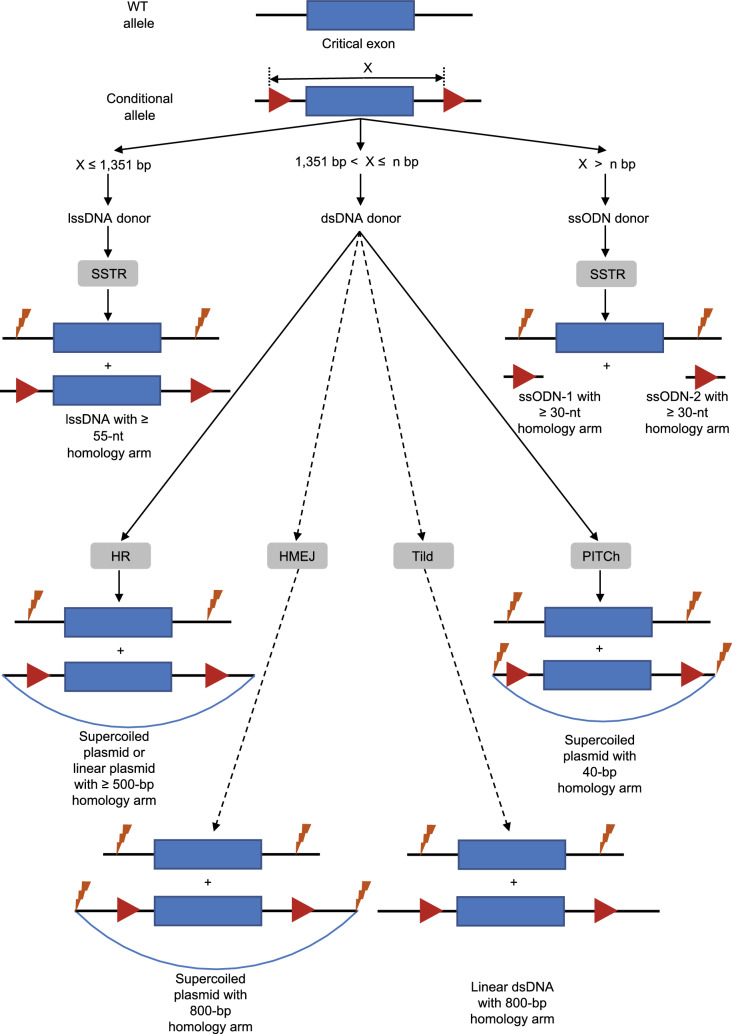
Conditional allele generation strategies in mammalian embryos. Four different strategies, indicated with solid lines, have been exploited to generate conditional allele depending on the distance between the two gRNA cleavage sites (defined as X). If X ≤ 1,351 bp, a lssDNA donor is recommended. If X > 1,351 bp, a dsDNA donor is recommended. The dashed line indicates two possible strategies, namely HMEJ and Tild, which remain to be tested in mammalian embryos. The upper limit of the conditional allele (n bp) that could be generated using a dsDNA donor remains to be investigated. Generating conditional allele using ssODN is inefficient, however, it might be used to generate very large conditional alleles (X > n bp). Intron, black line. Exon, blue box. gRNA cleavage site, yellow lightning bolt. LoxP, red triangle. Plasmid backbone, blue line. lssDNA, long single-stranded DNA. SSTR, single-stranded templated repair. dsDNA, double-stranded DNA. HR, homologous recombination. HMEJ, homology-mediated end joining. Tild, targeted integration with linearized dsDNA. PITCh, precise integration into the target chromosome. ssODN, single-stranded oligodeoxynucleotide

Although there are some cases that used two gRNAs and two ssODNs containing the LoxP sequence to generate conditional allele (referred herein as two-ssODN floxing method) (Fig. 2), generating a conditional allele using ssODN is inefficient (Yang et al., 2013; Pritchard et al., 2017; Lanza et al., 2018; Gurumurthy et al., 2019). In one comprehensive study, 56 genes were selected for generating the conditional allele by the two-ssODN floxing method (Gurumurthy et al., 2019). Among the 1,718 mouse pups generated, only 15 (0.87%) pups harbored the conditional allele (Gurumurthy et al., 2019). One major problem is that the two LoxP sites are integrated *in trans*, which means that they integrate into two different chromosomes instead of the same chromosome (Lanza et al., 2018; Gurumurthy et al., 2019). However, one advantage of the two-ssODN floxing method is that the distance between gRNA target sites (250–4,500 bp) did not affect the frequency of conditional allele generation (Lanza et al., 2018). This advantage is beneficial for generating a large conditional allele, making it feasible to knock out very large DNA fragment upon induction.

### HBR using lssDNA

The maximum length of lssDNA is 5,000 nt, after which they are prone to spontaneous breaks (Lanza et al., 2018). Compared with ssODN, lssDNA allows for larger fragment knock-ins and the efficient generation of conditional alleles in mouse and rat zygotes (Fig. 2 and Table S2) (Miura et al., 2015; Yoshimi et al., 2016). lssDNA uses a short homology arm (typically 55–329 nt at each end) to KI exogenous DNA (Table S2). Similar to ssODN, the distance between the modification and the Cas nuclease cleavage site should be as small as possible (Li et al., 2019). Combining two gRNAs and lssDNA, the intended mutation located between the two gRNA cleavage sites, which is remote from both gRNA cleavage sites (>30 bp), could be installed with a high efficiency (Table S2) (Codner et al., 2018). Utilizing lssDNA, Codner et al. generated a point-mutation mouse model whose intended mutation was 98-bp away from the gRNA cleavage site (Codner et al., 2018). However, one drawback of lssDNA is that it is more toxic than ssODN (Li et al., 2019).

lssDNA has been exploited for large fragment KI in cells and animal embryos, but not in human embryos (Miura et al., 2015; Yoshimi et al., 2016; Li et al., 2019). Using lssDNA as a donor results in a high GFP KI efficiency in human HEK-293T cells (5%–30%). Furthermore, longer homology arms improved the KI efficiency in HEK-293T cells, with 400–700 nt at each end as the optimal homology arm length (Li et al., 2019). In mouse zygotes, Rolen et al. were able to knock in 1,368-bp DNA fragment using lssDNA with a 90-nt homology arm at each end (Table S2) (Quadros et al., 2017). However, it remains unclear whether it is possible to generate larger DNA fragment KI (e.g., 5,000-nt DNA fragment) in animal zygotes using short homology arms (55–144 nt). To improve the efficiency of large DNA fragments KI, lssDNA with longer homology arms may be helpful (Li et al., 2019). PS-modifed lssDNA might also be able to improve the HBR efficiency, but it has not yet been tested in cells and mammalian embryos.

In addition, lssDNA can be used to generate a conditional allele. To the best of our knowledge, lssDNA has been successfully used to generate a 1,351-bp conditional allele (Table S2) (Codner et al., 2018). However, whether it is feasible to generate a larger conditional allele using lssDNA is unclear (Codner et al., 2018). Compared with ssODN, generating a conditional allele using lssDNA is much more efficient (Tables S1 and S2) (Codner et al., 2018; Gurumurthy et al., 2019). Because lssDNA harbors two LoxP sites, it requires only one recombination event, whereas the two-ssODN floxing method requires two simultaneous recombinations in the same chromosome (Quadros et al., 2017; Codner et al., 2018; Gurumurthy et al., 2019). Thus, lssDNA alleviates the challenge of integration *in trans* and improves the efficiency of generating a conditional allele. However, longer homology arms (100 nt vs. 60 nt) may be another important factor to enhance the efficiency (Tables S1 and S2) (Quadros et al., 2017).

However, producing lssDNA is more expensive and cumbersome than ssODN. The chemical synthesis of lssDNA is costly. The ivTRT method can generate a large amount of ≤2,000-nt lssDNA (>50 µg) (Li et al., 2019). However, the generated lssDNA will be contaminated by truncated lssDNA due to the poor processivity of the reverse-transcriptase (Mohr et al., 2013). The selection of ssDNA labeled with biotin and the strand-specific digestion of dsDNA require generating dsDNA by PCR, which may introduce sequence error (Li et al., 2019). Although the strand-specific digestion of dsDNA is more convenient than ivTRT, the amount of lssDNA generated is approximately 10-fold lower than that of the ivTRT method (Murgha et al., 2014; Yoshimi et al., 2016; Li et al., 2019). In addition, all the methods used to produce lssDNA may result in unexpected mutations in the DNA donor. Therefore, it is necessary to sequence the edited cells and animals to eliminate the effect of unexpected mutations.

### HBR using AAV DNA

In addition to ssODN and lssDNA, the linear single-stranded genomic DNA of AAV has been utilized as HBR templates. AAV is a single-stranded DNA virus that can be produced by transfecting virus-packaging cells with double-stranded AAV plasmids vectors.

Combining ZFN (or TALEN) with AAV serotype 6 (AAV6) donor, which contains homology arms flanking the insertion sequence, efficient targeted insertion could be induced in HSPCs and primary T cells (Sather et al., 2015; Wang et al., 2015). Later, it has been proven that combing CRISPR-Cas nuclease with AAV6 donor vector could also induce efficient KI in HSPCs (Dever et al., 2016; Pavel-Dinu et al., 2019). Intriguingly, the AAV serotype 1 (AAV1) donor vector induced a higher KI efficiency than conventional plasmid donor vector when combined with CRISPR-Cas nuclease in HEK-293T cells, U2OS cells, human dermal fibroblasts, and rat C6 cells (Gaj et al., 2017). Furthermore, the AAV donor is delivered to the liver, together with CRISPR-Cas nuclease, efficiently correcting the mutation in the hereditary tyrosinemia mouse model and hyperammonemia mouse model (Yang et al., 2016; Yin et al., 2016; Krooss et al., 2020). Delivering AAV donor and CRISPR-Cas nuclease into mouse zygotes results in efficient genome editing, including precise point mutation and KI of large DNA fragments (771–3,300 nt) (Table S3) (Yoon et al., 2018; Chen et al., 2019). However, whether it is feasible to generate a conditional allele using an AAV donor remains to be investigated.

One constraint of the AAV donor is its limited packaging capacity (~4,600 nt), making it difficult to insert larger gene coding sequences (e.g., *DMD* and *F8*) (Bak and Porteus, 2017). In general, the homology arm length of the AAV donor vector is 400–800 nt. Considering the two flanking 400-nt homology arms, the length of the inserted DNA fragments should be shorter than 3,800 nt (Bak and Porteus, 2017). By delivering two AAV donors together with CRISPR-Cas nuclease, it is feasible to insert 5,700-nt DNA in HSPCs and T cells (Bak and Porteus, 2017). However, whether it is possible to edit target genes in large mammalian animal and human embryos using AAV donors remains to be tested (Chen et al., 2011; Gaj et al., 2017). First, the AAV serotype that could infect large mammalian animal and human zygotes should be screened. Then, the editing efficiency using the AAV donor vector should be investigated. It is worth noting that using an AAV donor vector may impose new safety concerns about AAV infection.

HBR using ssODN and lssDNA has been shown to occur through the SSTR pathway (Renaud et al., 2016; Kan et al., 2017; Richardson et al., 2018). However, whether HBR using an AAV donor also occurs through the SSTR pathway remains to be elucidated.

SSTR is similar to the synthesis-dependent strand annealing (SDSA) pathway of HR (Kan et al., 2017; Yeh et al., 2019). While the SSTR pathway takes advantage of single-stranded DNA donors (Kan et al., 2017), the HR pathway utilizes a double-stranded DNA (dsDNA) donor. Unlike HR, SSTR is not Rad51-dependent (Bothmer et al., 2017; Richardson et al., 2018). Contrary to MMEJ and SSA, which are both independent of templated DNA synthesis using exogenous DNA donor as the template, SSTR and HR are characterized by strand invasion and subsequent strand extension using exogenous DNA donor as the template (Kan et al., 2017). However, the proteins involved in SSTR remain under-explored. It has been proposed that some proteins responsible for the MMEJ, SSA, and Fanconi anemia pathway may be involved in SSTR (Quadros et al., 2017; Richardson et al., 2018). To identify the proteins involved in the SSTR pathway, systemic loss-of-function screening is needed (Richardson et al., 2018). Knocking down either *Fanca* or *Fancd1,* genes involved in the Fanconi anemia pathway, was found to inhibit AAV-mediated KI in mouse cardiomyocytes (Kohama et al., 2020), suggesting that AAV-mediated KI occurs via the SSTR pathway.

**Figure 3:**
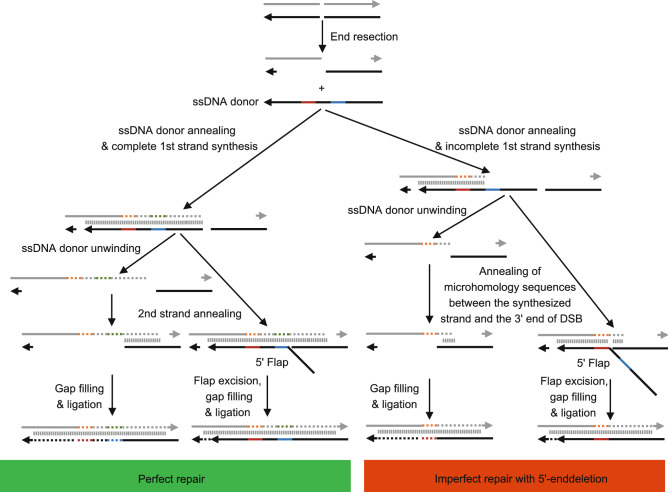
Potential mechanisms underlying perfect repair and imperfect repair via the SSTR pathway. DSB site, generated by CRISPR-Cas nuclease, undergoes end resection and generates 3′ overhangs. In the presence of ssDNA donor, the 3′ overhang anneals with the ssDNA donor and primes DNA synthesis, leading to complete or incomplete 1st strand synthesis. Complete 1st strand synthesis copies both the 3′-end edit (red line) and 5′-end edit (blue line). However, incomplete 1st strand synthesis only copies the 3′-end edit (red line). After 1st strand synthesis, the ssDNA donor is removed (or not removed) by helicase. The newly synthesized 1st strand anneals with homology sequences at the other end of the DSB site. If the ssDNA donor is removed, the DSB is repaired by gap filling and ligation. If the ssDNA donor is not removed, the annealing process generates a repair intermediate with a 5′ flap, which will be further repaired by flap excision, gap filling, and ligation, resulting in the retention of ssDNA donor at the target site. Whether SSTR results in the retention of ssDNA donor at the target site is still under debate. During incomplete 1st strand synthesis, the newly synthesized 1st strand searches the DSB site for microhomology sequences for hybridization, resulting in an imperfect repair without a 5′-end edit. The direction of the arrow represents the 5′ to 3′ direction. 3′-end edit, red line. 5′-end edit, blue line. Newly synthesized DNA, dashed line. SSTR, single-stranded templated repair. DSB, double-stranded break

It is noteworthy that genome editing using ssODN (or lssDNA) as HBR templates often displays deletion mutations at the 5′ end of the ssODN (or lssDNA) repair template (Renaud et al., 2016; Ge and Hunter, 2019). The 5′-end deletion may be due to the incomplete DNA synthesis and the MMEJ pathway-mediated deletion in cells and embryos (Fig. 3) (Renaud et al., 2016; Yoshimi et al., 2016). The elongation of the 5′ homology arm ameliorated the 5′-end deletion and enhanced the KI efficiency of ssODN and lssDNA, indicating that asymmetric ssODN and lssDNA with longer 5′ homology arm is better than symmetric ssODN (Renaud et al., 2016; Richardson et al., 2016; Yoshimi et al., 2016; Lanza et al., 2018; Wang et al., 2018). However, it remains to be investigated whether KI by AAV donor also shows a 5′ deletion propensity, similar to ssODN and lssDNA, when partial KI occurs (Renaud et al., 2016; Yoshimi et al., 2016; Canaj et al., 2019). 5′-end deletion propensity is an important feature of the SSTR pathway. If it does show 5′-end deletion propensity, KI by AAV donor might occur through SSTR pathway. In addition, whether elongating the 5′ homology arm will also enhance the precise KI efficiency of the AAV donor remains to be investigated.

Because the non-target strand is released earlier than the target strand after DNA cleavage by Cas9, Cas9 nuclease-induced HBR using ssODN donor showed donor strand bias at some sites (Richardson et al., 2016; Lanza et al., 2018). Combined with the Cas9 nuclease, ssODN complementary to the non-target strand displays higher HBR efficiency than ssODN, which is complementary to the target strand (Richardson et al., 2016; Lanza et al., 2018). In contrast to Cas9, Cas12a (e.g., AsCas12a and LbCas12a) exhibit a preference for ssODN complementary to the target strand (Wang et al., 2018). Whether the target strand is released earlier than the non-target strand after DNA cleavage by Cas12a remains to be investigated. In addition, whether CRISPR-Cas nuclease-induced HBR using lssDNA and AAV donor vector also displays such strand bias remains unclear.

## Homology-based repair using double-stranded DNA donor

dsDNA donors, including plasmid donor and linear dsDNA donor, are suitable for inserting large DNA fragments (> 3.8 kb), which is not possible using a single AAV vector (Table S4). HBR using dsDNA donors is less efficient than single-stranded DNA, but allows for larger fragment KI using CRISPR-Cas nuclease (up to 7.1 kb) (Table S4) (Menoret et al., 2015; Nakao et al., 2016; Quadros et al., 2017). dsDNA donors were used to insert a 7.1-kb DNA fragment at the target site in 3.1% (3/97) mouse pups (Table S4) (Nakao et al., 2016). HBR using a dsDNA donor occurs through three distinct pathways: homologous recombination (HR), single-stranded annealing (SSA), and microhomology-mediated end joining (MMEJ). Each pathway requires a different DNA donor vector construction strategy (Fig. 2).

HR takes advantage of exogenous dsDNA donors or endogenous homologous chromosomes as repair templates (Yoshimi et al., 2014). The exogenous dsDNA donors of HR could be either supercoiled or linear plasmids with long homology arms (Fig. 2 and Table S4) (Canaj et al., 2019). Compared with SSTR, HR requires much longer homology arms (>400 bp, typically 0.5–1 kb) (Fig. 2). In human cells, at least a 400-bp homology arm at each end is required for efficient nuclease-induced HR, and the lengthening of homology arms was found to increase the targeted insertion efficiency at some target sites (Hendel et al., 2014; Chu et al., 2015).

Successful gene KI or precise deletion has been achieved in mouse, rat, rabbit, pig, and human embryos by injecting supercoiled or linear plasmids into zygotes (Table S4). Linear plasmids showed a higher KI efficiency than the supercoiled plasmids, but also led to a higher random integration of donor plasmids (Menoret et al., 2015). In addition, conditional alleles could also be generated efficiently in mice, rats, and pigs (Table S4) (Lee and Lloyd, 2014). To the best of our knowledge, the 631-bp conditional allele, floxed by LoxP, is the largest allele generated by injecting plasmid donor and CRISPR-Cas nuclease (Table S4) (Ma et al., 2014). In theory, a larger conditional allele (up to 7.1 kb) could be generated, however, the upper limit (defined as n-bp) of the conditional allele, which could be generated using a plasmid donor, remains to be determined.

Although HR allows for the precise integration of large DNA fragments, the efficiency of HR varies among cell types and species. To increase the efficiency of KI, Yang et al. and Zhang et al. developed new KI strategies, which might occur via the SSA pathway (Yao et al., 2017; Zhang et al., 2017). In this strategy, supercoiled plasmids harboring synthetic gRNA target sites and two 800-bp homology arms were utilized as DNA donors (Fig. 2) (Yao et al., 2017; Zhang et al., 2017). The supercoiled plasmids were linearized *in vivo* and acted as repair templates (named homology-mediated end joining (HMEJ) strategy, Fig. 2) (Yao et al., 2017; Zhang et al., 2017).

SSA is very similar to MMEJ. First, DSBs and the dsDNA donor are processed via 5′-end resection to reveal long homology arms (>200 bp) (Liskay et al., 1987), which anneal to each other via base pairing (Fig. 1). Then, the gaps between the DSBs and dsDNA donor are filled and the breaks are sealed, leading to the insertion of exogenous DNA (Fig. 1). The enzyme involved in gap filling and break sealing has yet to be elucidated (Yeh et al., 2019).

A later study found that these supercoiled plasmids could be replaced with linear dsDNA with two 800-bp homology arms (named targeted integration with linearized dsDNA (Tild) strategy) (Fig. 2) (Yao et al., 2018b). Both types of DNA donors were capable of efficiently and precisely integrating exogenous DNA at the target site (Fig. 2) (Yao et al., 2018b). The HMEJ and Tild strategy displayed efficient HBR in mouse, monkey, and human embryos (Table S4) (Yao et al., 2017; Yao et al., 2018a, 2018b). However, whether it is possible to generate conditional allele using HMEJ and Tild strategy has not yet been studied, although it is feasible in principle (Table S4) (Yao et al., 2017, 2018a, 2018b).

The construction of plasmid donors with long homology arms is challenging and time-consuming, especially when the target region contains a high GC content or repetitive sequences. In addition, because of the long homology arm, it is difficult to apply PCR and sequencing strategies to screen out the edited cells or animals. A laborious Southern blot assay should be performed to identify and confirm the edited cells or animals.

Precise integration into the target chromosome (PITCh) strategy uses supercoiled plasmids harboring synthetic gRNA target sites and two microhomology arms as the repair template (Nakade et al., 2014). Constructing a PITCh plasmid donor is much more convenient than the HR and SSA plasmid donors because of its short homology arm (5–40 bp) (Nakade et al., 2014). After delivering the supercoiled plasmids and CRISPR-Cas nuclease into the cells, both the supercoiled plasmids and the target site are cleaved. The microhomology arms then aid the integration of the DNA, flanked by the two microhomology arms, through the MMEJ pathway (Fig. 2). First, the DSB and the dsDNA donor are processed by 5′-end resection to reveal a short homology arm, resulting in annealing via base pairing (Fig. 1). Then, DNA polymerase θ binds the annealed products and fills the gaps between the DSB and dsDNA donor by templated synthesis (Fig. 1). Finally, the DNA breaks are sealed by DNA ligase I or DNA ligase III, resulting in the targeted insertion of exogenous DNA (Fig. 1) (Yeh et al., 2019).

Unlike HR, which is active during the late S/G_2_ phases, MMEJ is active during the G_1_/early S phases. Successful targeted integration by PITCh has been reported in silkworm embryos and zebrafish embryos, whose HR efficiency is low (Nakade et al., 2014). Using the PITCh strategy, a 5-kb DNA fragment was successfully inserted at the target site in 12% (3/25) mouse zygotes (Table S4) (Aida et al., 2016). The ectopic expression of some MMEJ-related genes (e.g., *EXO1*, *LIG3*, *PARP1*, *NBS1*, *FEN1*, *BLM*, and *MRE11A*) enhanced MMEJ efficiency in human cells, whereas the ectopic expression of HR-related genes (e.g., *RAD51*) and SSA-related genes (e.g., *RAD52*) suppressed MMEJ (Aida et al., 2016). Exonuclease 1 (EXO1) is a 5′–3′ exonuclease involved in the end resection of DSB. Co-injected with EXO1 nuclease, the KI efficiency of PITCh increased about 3-fold in mouse zygotes (Table S4) (Aida et al., 2015, 2016; Hisano et al., 2015). Furthermore, a conditional knockout mouse model was generated efficiently (33.3%) via the PITCh strategy (Table S4) (Aida et al., 2016). Therefore, the PITCh strategy allows for both the efficient generation and convenient identification of large fragment KI animal models.

Although HBR efficiency varied site by site, the above four strategies (HR, HMEJ, Tild, and PITCh) showed different HBR efficiencies in mammalian embryos. Compared with conventional HR using supercoiled plasmid, Tild achieved a 6.4-fold higher HBR efficiency in human embryos (Yao et al., 2018b). Comparing the HBR efficiency of PITCh and HMEJ revealed that HMEJ is much more efficient (1.9–3.7-fold) than PITCh at three target sites (*Actb*, *Dbh*, and *Sox2*) in mouse embryos (Table S4) (Yao et al., 2017). Moreover, Tild (targeted integration with linearized dsDNA) showed a higher (1.6–3.3-fold) HBR efficiency than HMEJ (homology-mediated end joining) at three sites (*Cdx2*, *Actb*, and *Sp8*) in mouse embryos (Table S4) (Yao et al., 2018b). Thus, the HBR efficiency in mammalian embryos may follow the order: Tild > HMEJ > PITCh ≥ HR. Based on current data, the Tild and HMEJ strategies may be the first choice for large DNA fragment knock-in in large mammalian animal and human embryos.

## Enhanced homology-based repair through tethering of DNA donor

**Figure 4:**
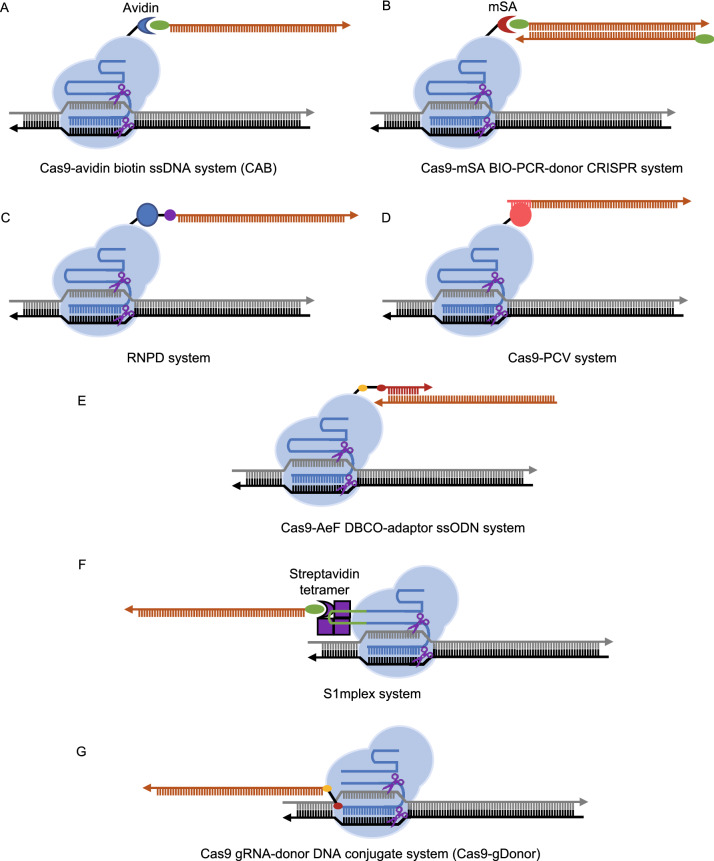
Different methods to recruit donor DNA to target site. (A) Cas9-avidin biotin ssDNA (CAB) system. Biotin, green oval. (B) Cas9-mSA BIO-PCR-donor CRISPR system. mSA, monomeric streptavidin. (C) The ribonucleoprotein DNA (RNPD) system. SNAP peptide, blue circle. O^6^-benzylguanine (BG), purple circle. Covalent bond between SNAP peptide and O^6^-benzylguanine (BG), black line. (D) The Cas9-PCV system. PCV, porcine circovirus 2 Rep protein, is shown with pink circle. PCV recognition sequence, pink line. (E) Cas9-AeF DBCO-adaptor ssODN system. AeF, noncanonical amino acid 4-(2-azidoethoxy)-l-phenylalanine. Azide group of AeF, golden oval. DBCO, red oval. Adaptor, red line. (F) S1mplex system. Chimeric RNA, composed of gRNA and S1m aptamer, recruits biotin-labeled ssDNA donor to the target site by interacting with the streptavidin tetramer (purple box). S1m aptamer, green line. (G) Guide RNA donor DNA conjugate (gDonor) system. Azide group, golden oval. DBCO, red oval. gRNA, blue line. DNA donor, orange line. The direction of the arrow represents the 5′ to 3′ direction

As HBR requires the presence of a DNA donor at the target site, many efforts have been devoted to increasing the concentration of DNA donors at target sites to enhance HBR. Recently, several methods have been developed to recruit DNA donors to target sites by tethering DNA donors to the gRNA-Cas9 complex (Fig. 4).

The Cas9-avidin biotin ssDNA (CAB) system takes advantage of Cas9-Avidin fusion protein and ssDNA labeled with biotin at the 5′ end (Fig. 4A) (Ma et al., 2017b). The interaction between avidin and biotin recruits a DNA donor to the target site, resulting in an increased HBR efficiency (2–5-fold) in human cells (Ma et al., 2017b). Furthermore, the CAB system enhanced HBR efficiency in mouse embryos by 3-fold compared with conventional CRISPR-Cas9 nuclease (Ma et al., 2017b). A similar system, based on the interaction between monomeric streptavidin (mSA) and biotin, was developed. Unlike the CAB system, the Cas9-mSA BIO-PCR-donor CRISPR system utilized biotin-labeled dsDNA donor as a repair template (Fig. 4B) (Gu et al., 2018; Roche et al., 2018). The biotin-labeled dsDNA donor was generated by PCR amplification using primers with 5′-biotin modification. The Cas9-mSA BIO-PCR-donor CRISPR system enhanced the HBR efficiency in human cells and in 2-cell mouse embryos (1.6–3.8-fold) (Gu et al., 2018; Roche et al., 2018).

In addition to avidin–biotin non-covalent interactions, other protein–substrate covalent interactions have been used to recruit DNA donors. The ribonucleoprotein DNA (RNPD) system is composed of gRNA, Cas9-SNAP fusion protein, and O^6^-benzylguanine (BG)-labeled ssODN (Savic et al., 2018). SNAP peptide could bind the O^6^-benzylguanine (BG)-labeled ssODN covalently, recruiting ssODN to the target site. The RNPD system enhanced the HBR efficiency in human HEK-293T cells (7–24-fold), K562 cells (17-fold), and mouse embryonic stem cells (mESCs) (2–6-fold) (Savic et al., 2018). Intriguingly, an increase in the ssODN concentration in the nucleus was sufficient to enhance HBR efficiency (Savic et al., 2018). Similarly, the Cas9-PCV system is based on the covalent interaction between the porcine circovirus 2 (PCV) Rep protein and its recognition DNA sequence (Aird et al., 2018). ssODN with a PCV recognition sequence is recruited to the target site by the gRNA-Cas9-PCV complex, resulting in enhanced HBR efficiency (Aird et al., 2018).

Recently, a method based on noncanonical amino acid (ncAA) modified Cas9 was developed (Ling et al., 2020). In this Cas9-AeF DBCO-adaptor ssODN system, the G1367 residue of Cas9 was replaced with a ncAA named 4-(2-azidoethoxy)-l-phenylalanine (AeF). Purified Cas9-AeF proteins were then incubated with gRNA, DBCO-modified ssDNA adaptor, and a ssODN donor. The azide group of AeF reacts with the alkyne of dibenzylcyclooctyne (DBCO), linking DBCO-modified ssDNA adaptors to Cas9. The linked ssDNA adaptor then recruits the ssODN donor by base pairing, tethering ssODN to the gRNA-Cas9 complex. Transfecting the Cas9-AeF DBCO-adaptor ssODN system into human cells led to a 10-fold increase in HBR efficiency (Ling et al., 2020). Moreover, injecting the Cas9-AeF DBCO-adaptor ssODN system into mouse embryos also led to a 2.3-fold increase in HBR efficiency (Ling et al., 2020).

Like Cas9 protein, gRNA can also be used to recruit DNA donors. The S1m aptamer, an RNA fragment capable of binding streptavidin tetramer, is installed in the first stem loop of the gRNA. Biotin-labeled ssDNA donor interacts with streptavidin tetramer, which is recruited to the gRNA-Cas9 complex by the S1m aptamer (Fig. 4F) (Carlson-Stevermer et al., 2017). Delivering this S1mplex system into human cells led to an enhanced HBR/indel frequency ratio (2.7–18.4-fold); however, this led to decreased absolute HBR efficiency (Carlson-Stevermer et al., 2017). In addition, donor DNA can be directly conjugated with guide RNA. In the Cas9 gRNA-donor DNA conjugate system, donor DNA is conjugated with the 5′ end of crRNA in the crRNA-tracrRNA dimer, forming a gRNA-donor DNA conjugate (gDonor) (Lee et al., 2017). Purified Cas9 protein is able to assemble with gDonor *in vitro* (Lee et al., 2017) (Fig. 4G). After delivering this assembled complex into human cells, a obvious increase in HBR efficiency was observed (Lee et al., 2017).

Of the seven systems mentioned above, only three systems (CAB system, Cas9-mSA BIO-PCR-donor CRISPR system, and Cas9-AeF DBCO-adaptor ssODN system) have been proven to be effective in mouse embryos. It would be interesting to compare the efficiency of all seven systems in mammalian embryos under the same circumstances. These results will help us to determine the most efficient and safe system for mammalian embryo genome editing.

## Homology-based repair using endogenous homologous DNA

In addition to exogenous DNA donors, endogenous homologous DNA can be utilized as an HBR template in mouse and human tripronuclear zygotes (Wu et al., 2013; Liang et al., 2015). Our group found that human tripronuclear zygotes prefer endogenous homologous DNA (*HBD* gene) to ssODN donors while editing *HBB* gene in human tripronuclear zygotes (Liang et al., 2015). The *HBD* gene used as a repair template may be from either the paternal or maternal chromosome (Liang et al., 2015). Later, Mitalipov et al. found that human diploid zygotes prefer to use the wild-type maternal chromosome than ssODN as repair templates (Ma et al., 2017a). However, owing to the rapid cleavage of target DNA by CRISPR-Cas nuclease and the spatial distance between paternal and maternal chromosomes in one-cell embryos, whether the maternal chromosome could be utilized as repair templates remains a topic of controversy (Ma et al., 2017a, 2018; Adikusuma et al., 2018; Egli et al., 2018; Reichmann et al., 2018). An alternative interpretation of Mitalipov’s data is that the paternal allele failed to be amplified due to the loss of segmental paternal chromosome as a result of large DNA fragment deletions, segmental paternal chromosome gain due to DNA fragment duplication, complete paternal chromosome loss, or translocations (Adikusuma et al., 2018; Egli et al., 2018; Alanis-Lobato et al., 2020; Zuccaro et al., 2020). Recently, Egli et al. found that templated repair using maternal chromosomes may occur in two-cell human embryos when the paternal and maternal genomes are in the same nucleus. However, its efficiency is relatively low (~7%) (Zuccaro et al., 2020). At the one-cell stage, templated repair using maternal chromosome did not occur due to separation of the male and female pronucleus (Zuccaro et al., 2020). Moreover, they found that even a single CRISPR-Cas nuclease-induced cut could lead to frequent loss of targeted chromosomes by destabilizing the entire chromosome in human early embryos, resulting in the failure of targeted DNA amplification using PCR (Zuccaro et al., 2020). It is noteworthy that these findings also underscore the risk of large fragment deletion and aneuploidy while editing the human embryo genome using CRISPR-Cas nuclease (Zuccaro et al., 2020). Further mechanistic and methodological studies are needed to develop techniques to eliminate large fragment deletions and aneuploidy while editing the human embryo genome using CRISPR-Cas nuclease.

HBR using endogenous homologous DNA template can reduce the efficiency of generating intended genome modifications using exogenous DNA donors. However, it may also allow to correct gene mutations through templated repair using endogenous homologous DNA template (Liang et al., 2015). Thus, depending on the purpose of genome editing, HBR using endogenous homologous sequences could be either beneficial or harmful.

## RNA-templated DNA repair: prime editing

In addition to DNA, RNA can be utilized as repair templates. Recently, Liu et al. developed prime editors, consisting of prime editor (PE) protein and prime editing guide RNA (pegRNA), to edit the genome without inducing DNA DSB (Anzalone et al., 2019). PE protein is composed of Cas9 nickase (Cas9n-H840A) and M-MLV reverse transcriptase variant (M-MLV D200N + L603W + T330P + T306K + W313F). pegRNA is comprised of four parts: a guide sequence, a gRNA backbone, a reverse transcription (RT) template, and a primer binding site (PBS). Under the guidance of pegRNA, the PE:pegRNA complex binds the target strand at the target site, resulting in the displacement of the non-target strand. Then, the Cas9n-H840A RuvC nuclease domain of the PE protein cleaves the non-target strand, exposing a 3′-hydorxyl group on the non-target strand. The cleaved and displaced non-target strand will hybridize with the PBS of pegRNA by base pairing, and the 3′-hydroxyl group on this strand will be used to prime reverse transcription by the MMLV reverse transcriptase domain. Thus, the edit encoded in the RT template of the pegRNA will be copied into the target site. The newly synthesized DNA will displace the wild-type sequence at the target site, resulting in a 5′-flap, which is cleaved and sealed by the DNA repair enzyme.

Based on this strategy, prime editors have been used to generate precise point mutations, small deletions (≤ 80 bp), and insertions (≤ 40 bp) in HEK-293T cells (Anzalone et al., 2019). However, for unknown reasons, the efficiency of the prime editor is very low in human iPSCs and mouse embryos (Liu et al., 2020; Surun et al., 2020). Therefore, further study is needed to improve the efficiency of prime editors in mammalian embryos.

## Conclusion and perspectives

**Figure 5:**
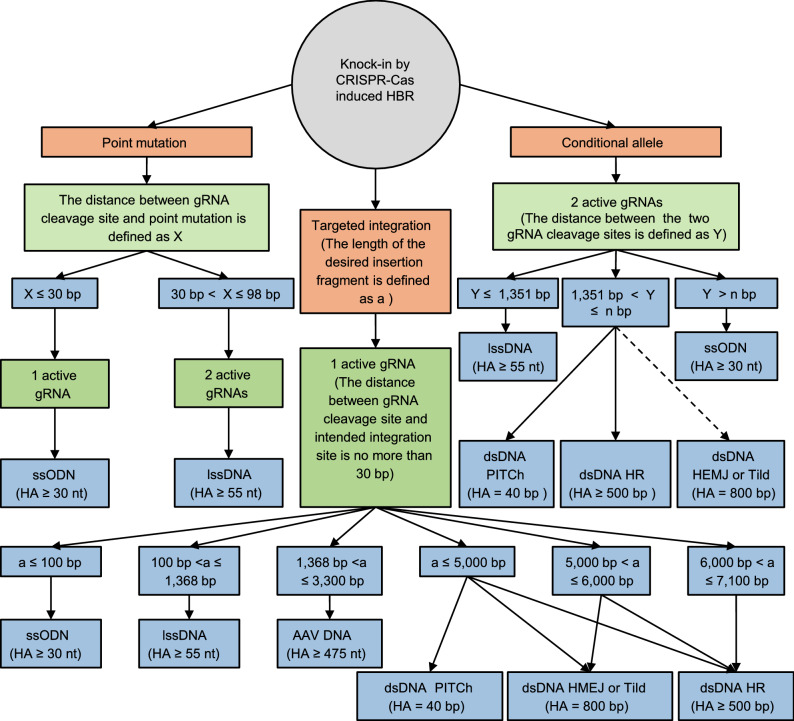
Decision tree for the selection of the HBR donor vector. HBR donor vectors should be designed based on several critical considerations, including the desired knock-in type (point mutation, targeted integration and conditional allele), the distance between gRNA cleavage site and the desired edit site (X), the length of the desired insertion fragment (a), and the distance between the two gRNA cleavage sites (Y). For point mutations, if X ≤ 30 bp, a ssODN donor is recommended. However, if 30 < X ≤ 98 bp, a lssDNA donor is recommended. For targeted integration, if X ≤ 30 bp, strategies that used one active gRNA are recommended. If a ≤ 100 bp, ssODN is recommended. If the length of the desired insertion fragment increases, other strategies are recommended, as shown in this figure. For targeted integration, if X > 30 bp, strategies similar to conditional allele generation should be used. For conditional allele generation, two active gRNAs should be identified at first. If the distance between the two active gRNAs (Y) is no more than 1,351 bp, lssDNA is recommended. The upper limit of the conditional allele (n bp) that could be generated using dsDNA remains to be investigated. If 1,351 < Y ≤ n bp, a dsDNA donor is recommended (e.g., PITCh and HR). Although HEMJ and Tild have not yet been applied for generating conditional allele in mammalian embryos, HEMJ and Tild could be used to generate conditional allele in theory. Although generating conditional alleles using ssODN is inefficient, it could be used to generate very large conditional allele (Y > n bp). The solid lines indicate strategies that have been demonstrated in mammalian embryos. The dashed line indicates possible strategies (HEMJ and Tild) that remain to be tested in mammalian embryos

ssDNA and dsDNA donors have both advantages and disadvantages. For example, ssDNA may introduce sequence errors in homology arms (ssODN and lssDNA), LoxP sites (ssODN and lssDNA), and the LoxP floxed region (ssODN and lssDNA) (Lanza et al., 2018). ssDNA could be used to generate KI mice by electroporation-based delivery (e.g., zygote electroporation and iGONAD), whereas successful genome editing using dsDNA donor delivered by zygote electroporation has yet to be reported. To date, dsDNA donors have been delivered into zygotes by microinjection (Remy et al., 2017; Miyasaka et al., 2018; Ohtsuka et al., 2018). Therefore, delivering ssDNA donors into zygotes is much more convenient than delivering dsDNA donors. In addition, dsDNA donors often lead to a higher cell toxicity and more off-target integration (Chen et al., 2011). To provide a guideline for the design of HBR DNA donors for mammalian embryo genome editing, the DNA donor designs and editing outcomes have been summarized in Table 1. Furthermore, a decision tree illustrating the main considerations for DNA donor design is provided in Fig. 5.

**Table 1:** Summary of donor DNA design and editing outcomes in mammalian embryos.

Donor DNA	5′ homology arm	3′ homology arm	Major editing type		
			Point mutation	Insertion	Conditional allele
ssODN	≥30 nt	≥30 nt	OK	≤100 nt	Not efficient
lssDNA	≥55 nt	≥55 nt	OK	≤1,368 nt	OK
AAV	≥475 nt	≥475 nt	OK	≤3,300 nt	Unknown
Supercoiled plasmid^a^	≥500 bp	≥500 bp	Unnecessary	≤7,100 bp	OK
Supercoiled plasmid^b^	800 bp	800 bp	Unnecessary	~800 bp	Unknown
Supercoiled plasmid^c^	40 bp	40 bp	Unnecessary	≤5,000 bp	OK
Linear plasmid^a^	≥500 bp	≥500 bp	Unnecessary	≤7,100 bp	OK
Linear dsDNA^d^	800 bp	800 bp	Unnecessary	≤6,000 bp	Unknown

Note: It is unnecessary to used plasmids and linear dsDNA to generate point mutation mammals. a, HR. b, HMEJ. c, PITCh. d, Tild

Recently, Iyer et al. produced long circular single-stranded DNA (cssDNA) by superinfecting *E*. *coli* cells containing phagemids with VCSM13 helper phages (Iyer et al., 2019). The DNA replication machinery provided by the helper phage recognizes the f1 origin of the phagemids, producing phages with circular single-stranded DNA. These phages are then harvested to purify the cssDNA. Long cssDNA donors have been used as HBR templates in human HEK-293T cells and K562 cells. Compared with the linear ssDNA donor, long cssDNA results in a much higher HBR efficiency (Iyer et al., 2019). Exploring whether long cssDNA also enhance HBR in mammalian embryos could provide new avenues for mammalian embryo genome editing.

In addition to the DNA donor, the cutting efficiency of CRISPR-Cas nuclease is a major factor that affects HBR efficiency (Lanza et al., 2018). Therefore, before designing DNA donors, highly active gRNAs should be screened by delivering them together with CRISPR-Cas nuclease into cells or embryos. In addition, changing the formulation of CRISPR-Cas nuclease could also increase its cutting efficiency. The ctRNP (crRNA + tracrRNA + Cas9 protein) complex could result in a higher KI efficiency than the Cas9-mRNA/gRNA mixture and gRNP (gRNA + Cas9 protein) complex (Quadros et al., 2017).

Additionally, controlling the time of CRISPR-Cas nuclease and DNA donor delivery also helps to increase HBR efficiency and reduce mosaicism (Ma et al., 2017a; Gu et al., 2018). It has been reported that microinjection at the two-cell stage increased the KI efficiency over 10-fold compared to that of the one-cell stage in mice (Gu et al., 2018). However, whether injecting CRISPR-Cas nuclease and DNA donor into the S/G_2_ phase (PN3-PN5) zygotes results in a higher editing efficiency than G1 phase (PN0-PN2) zygotes remains to be determined (Wossidlo et al., 2011).

In addition, the specificity of gRNA is also an important consideration. Cas9 nickase (Cas9n), which only cleaves one strand of the DNA double helix, has an increased specificity but is not routinely used for KI because of its low efficiency (Lee and Lloyd, 2014; Cornu et al., 2017; Kan et al., 2017). It is worth noting that both ssDNA and dsDNA donors are able to integrate randomly into the genome, particularly at off-target sites (Quadros et al., 2017; Lanza et al., 2018; Li et al., 2019). Therefore, it is important to check the random integration events of DNA donors in F1-generation animals.

In addition to CRISPR-Cas nuclease-mediated HBR, base editors have been used to efficiently generate point mutations without inducing DNA DSB in mammalian and human embryos (Kim et al., 2017b; Liang et al., 2017, 2018; Liu et al., 2018b, 2018c; Ryu et al., 2018; Yang et al., 2018; Anzalone et al., 2020). Cytidine base editor (CBE), which is composed of cytidine deaminase, Cas9n-D10A, and a uracil DNA glycosylase inhibitor (UGI) fusion protein, is able to catalyze C-to-T conversion in the activity window under the guidance of gRNA (Komor et al., 2016), while C-to-G base editor (CGBE), which is composed of cytidine deaminase and Cas9n-D10A fusion protein, can catalyze C-to-G conversion in the activity window (Kurt et al., 2020; Zhao et al., 2020b). Similarly, an adenine base editor (ABE), which is composed of adenine deaminase and Cas9n-D10A fusion protein, catalyzes A-to-G conversion in the activity window (Gaudelli et al., 2017). When editing the genome of mammalian and human embryos using base editors, care must be taken with the off-target DNA and RNA mutations (Kim et al., 2017a, 2017c, 2019; Grunewald et al., 2019a; Jin et al., 2019; Liang and Huang, 2019; Liang et al., 2019a, 2019b; Zhou et al., 2019; Zuo et al., 2019). High-fidelity base editor variants with lower off-target effects should be used (Liang et al., 2017; Grunewald et al., 2019b; Liang and Huang, 2019; Liang et al., 2019a; Rees et al., 2019; Zhou et al., 2019; Doman et al., 2020; Yu et al., 2020; Zuo et al., 2020). A recent review by Anzalone et al. is an excellent guide to choose the appropriate base editors for genome editing (Anzalone et al., 2020).

Moreover, the targeted insertion of large DNA fragments by CRISPR-associated transposases has been reported in *E*. *coli* (Klompe et al., 2019; Strecker et al., 2019). However, whether these CRISPR-associated transposases can lead to targeted insertion in eukaryotic cells has yet to be elucidated. Thus, an in-depth study of CRISPR-associated transposases may enable the targeted insertion of large DNA fragments in eukaryotic cells and mammalian embryos.

The advent of genome editing tools has enabled the generation of genome-edited mammalian animals, which will not only help to improve our understanding of fundamental biology underlying life and disease, but also promote advances in livestock farming. These tools can be used for the repair disease mutations *in situ*, with the potential to make incurable diseases curable and promote the development of precision medicine in the future.

## Supplementary Information

Below is the link to the electronic supplementary material.

Electronic supplementary material 1 (PDF 310 kb)
