# Supplementary material for: Homology-based repair induced by CRISPR-Cas nucleases in mammalian embryo genome editing
Source: Protein Cell. 2021 May 4;13(5):316–35. doi: 10.1007/s13238-021-00838-7 (PMC9008090; doi:10.1007/s13238-021-00838-7)
Supplement: Supplementary file 1 — Electronic supplementary material 1 (PDF 310 kb) [file 13238_2021_838_MOESM1_ESM.pdf]

Table S1 HBR by CRISPR-Cas nuclease and ssODN in mammalian zygotes

Donor DNA	5' homology arm (nt)	3' homology arm (nt)	Insertion (nt)	Species	Target gene	Efficiency in embryo (Edited/total embryos)	Efficiency in pups (Edited/total pups)	Reference
ssODN	62	62	2-nt mutation	Mouse	*Tet1*	28.5% (4/14)	70% (7/10)	(Wang et al., 2013)
ssODN	62	62	2-nt mutation	Mouse	*Tet2*	50% (7/14)	80% (8/10)	(Wang et al., 2013)
ssODN	43	23	1-nt insertion and 2-nt mutations	Mouse	*Crygc*	-	11.1% (3/27)	(Wu et al., 2013)
ssODN	67	44	1-nt mutation	Mouse	*Sf-1*	-	17.6% (9/51)	(Inui et al., 2014)
ssODN	77	32	1-nt mutation	Mouse	*Sf-1*	-	29.6% (16/54)	(Inui et al., 2014)
ssODN	51	66	2-nt mutation	Mouse	*Tex15*	-	56.2% (9/16)	(Singh and Schimenti, 2015)
ssODN	65	59	2-nt mutation	Mouse	*Mlh1*	-	29.1% (7/24)	(Singh and Schimenti, 2015)
ssODN	59	63	3-nt mutation	Mouse	*Cdk2*	-	44.4% (10/27)	(Singh and Schimenti, 2015)
ssODN	~60	~60	1-nt mutation	Mouse	*Syne1-E646*	-	53.3% (8/15)	(Ma et al., 2017a)
ssODN	~60	~60	1-nt mutation	Mouse	*Nexn-R274*	-	53.3% (8/15)	(Ma et al., 2017a)
ssODN	~60	~60	1-nt mutation	Mouse	*Tax1bp3*	-	50% (7/14)	(Ma et al., 2017a)

ssODN	~60	~60	1-nt mutation	Mouse	*Flnc*	-	60% (3/5)	(Ma et al., 2017a)
ssODN	~60	~60	2-nt mutation	Mouse	*Tln*	-	66.7% (2/3)	(Ma et al., 2017a)
ssODN	~60	~60	2-nt mutation	Mouse	*Syne1-R257*	-	35.2% (6/17)	(Ma et al., 2017a)
ssODN	~60	~60	3-nt mutation	Mouse	*Syne1-W463*	-	33.3% (3/9)	(Ma et al., 2017a)
ssODN	97	100	1-nt mut ation	Mouse	*Fgfr3G1120A*	-	25.7% (9/35)	(Miao et al., 2019)
ssODN	48	50	1-nt mut ation	Mouse	*Fgfr3G1120A*	-	22% (9/41)	(Miao et al., 2019)
ssODN	~60	~60	5-nt mut ation	Mouse	*Cnn1*	-	16.6% (3/18)	(Han et al., 2015)
ssODN	80	87	5-nt mut ation	Mouse	*Dmd*	-	8.7% (2/23)	(Long et al., 2014)
ssODN	60	57	3-nt mut ation	Mouse	*Spp1*	-	25% (2/8)	(Nakagawa et al., 2016)
ssODN	~50	~50	12	Mouse	*Fah*	-	55.6% (5/9)	(Li et al., 2014)
ssODN	60	60	30	Mouse	*Nle*	39.6% (21/53)	-	(Raveux et al., 2017)
ssODN	60	60	40	Mouse	*Tet1*	40% (6/15)	-	(Yang et al., 2013)
ssODN	60	60	40	Mouse	*Tet2*	53.3% (8/15)	-	(Yang et al., 2013)
ssODN	60	60	42	Mouse	*Sox2*	43.8% (7/16)	34.3% (12/35)	(Yang et al., 2013)
ssODN	60	60	40	Mouse	*Mecp2* (L2)	-	45.9% (45/98)	(Yang et al., 2013)
ssODN	60	60	40	Mouse	*Mecp2* (R1)	-	25.5% (25/98)	(Yang et al., 2013)
ssODN	50	50	40	Mouse	*Rosa26*	9.5% (2/21)	-	(Renaud et al., 2016)

PS-modified ssDON	50	50	40	Mouse	*Rosa26*	62.5% (5/8)	-	(Renaud et al., 2016)
ssODN	30	30	70	Mouse	*H1c*	16.7% (2/12)	-	(Zhang et al., 2016)
ssODN	30	30	70	Mouse	*H1c*	16.7% (2/12)	-	(Zhang et al., 2016)
ssODN	60	60	78	Mouse	*Rosa26-cr2*	-	33.3% (11/33)	(Quadros et al., 2015)
ssODN	60	60	78	Mouse	*Rosa26-cr4*	-	36.4% (8/22)	(Quadros et al., 2015)
ssODN	39	56	87	Mouse	*Sidt2*	-	23.1% (3/13)	(Ge and Hunter, 2019)
ssODN (Two-ssODN floxing method)	60	60	40 (LoxP+ RE site)	Mouse	*Mecp2*	-	16.3% (16/98)	(Yang et al., 2013)
ssODN (Two-ssODN floxing method)	60	60	40 (LoxP+ RE site)	Mouse	*Mecp2*	0% (0/149)	-	(Gurumurthy et al., 2019)
ssODN (Two-ssODN floxing method)	61	61	34 (LoxP)	Mouse	*Cluster A*	-	5.5% (3/55)	(Pritchard et al., 2017)
ssODN (Two-ssODN floxing method)	61	61	34 (LoxP)	Mouse	*Gene B*	-	2.9% (1/35)	(Pritchard et al., 2017)
ssODN (Two-ssODN floxing method)	61	61	34 (LoxP)	Mouse	*Gene C*	-	18.2% (2/11)	(Pritchard et al., 2017)
ssODN	61	61	34	Mouse	*Gene D*	-	5% (1/20)	(Pritchard et al.,

(Two-ssODN floxing method)			(LoxP)					2017)
ssODN (Two-ssODN floxing method)	61	61	34 (LoxP)	Mouse	*Cluster E*	-	0% (0/35)	(Pritchard et al., 2017)
ssODN (Two-ssODN floxing method)	61	61	34 (LoxP)	Mouse	*Gene F*	-	0% (0/36)	(Pritchard et al., 2017)
ssODN	39	40	1-nt mutation	Rat	*Thy1*	-	41.9% (13/31)	(Yoshimi et al., 2016)
ssODN	38	40	1-nt mutation	Rat	*Tyr*	36.8% (14/38)	-	(Yoshimi et al., 2014)
ssODN	45	45	1-nt mutation	Rat	*Cftr*	-	24.6% (16/65)	(Renaud et al., 2016)
PS-modified ssDON	45	45	1-nt mutation	Rat	*Cftr*	-	31.7% (13/41)	(Renaud et al., 2016)
ssODN	~50	~50	3-nt mutation	Rat	*Ephx2*	-	24.6% (16/65)	(Remy et al., 2017)
ssODN	44	43	6-nt mut ation	Rat	*Anks3*	-	18.2% (4/22)	(Menoret et al., 2015)
ssODN	~50	~50	5-nt mutation	Rat	*FlnA*	-	24.6% (16/65)	(Remy et al., 2017)
ssODN	29	30	40	Rat	*Tgr5*	-	16.7% (2/12)	(Shao et al., 2014)
ssODN	~40	~40	3-nt mutation	Rabbit	*Tyr*	-	29.4% (5/17）	(Song et al., 2018)
ssODN	54	50	3-nt mutation	Pig	*Sox10*	-	80% (4/5)	(Zhou et al., 2016)
ssODN	75	75	40	Pig	*Zbed6*	20% (2/10)	-	(Park et al., 2017)

ssODN	50	50	100	Pig	*Col1a*	100% (5/5)	58.3% (7/12)	(Park et al., 2017)
ssODN	51	51	6-nt mutation	Sheep	*Otof*	-	61.5% (8/13)	(Menchaca et al., 2020)
ssODN	50	50	3-nt mutation	Bovine	*Dnmt3a*	22.2% (10/45)	-	(Lamas-Toranzo et al., 2020)
ssODN	49	60	6-nt mutation	Human	*HBB*	9.7% (3/31)	-	(Liang et al., 2015)
ssODN	44	45	32-nt deletion	Human	*CCR5*	5% (1/20)	-	(Kang et al., 2016)
ssODN	44	40	6-nt mutation	Human	*G6PD site 1*	20% (2/10)	-	(Tang et al., 2017)
ssODN	34	43	6-nt mutation	Human	*G6PD site 3*	100% (2/2)	-	(Tang et al., 2017)
ssODN	92	66	3-nt deletion	Human	*HBB site 1*	10% (1/10)	-	(Tang et al., 2017)
ssODN	45	42	3-nt mutation	Human	*HBB site 2*	25% (1/4)	-	(Tang et al., 2017)

RE, Restriction enzyme recognition site.

Table S2 HBR by CRISPR-Cas nuclease and lssDNA in mammalian zygotes

5' homology arm (nt)	3' homology arm (nt)	Insertion (nt)	Species	Target gene	Efficiency in embryo (Edited/total embryos)	Efficiency in pups (Edited/total pups)	Reference
100	100	5-nt mutation	Mouse	*Gckr*	-	36.4% (8/22)	(Codner et al., 2018)
100	100	5-nt mutation	Mouse	*Rims1*	-	10.3% (4/39)	(Codner et al., 2018)
77	80	96	Mouse	*Sidt1*	-	100% (32/32)	(Ge and Hunter, 2019)

80	80	87	Mouse	*Sidt2*	-	37.5% (12/32)	(Ge and Hunter, 2019)
55	55	186	Mouse	*eEF2-TS2*	10% (1/10) - 66.7% (4/6)	-	(Miura et al., 2015)
55	55	324	Mouse	*eEF2-TS1*	50% (3/6)	-	(Miura et al., 2015)
55	55	324	Mouse	*eEF2-TS2*	83.3% (5/6)	-	(Miura et al., 2015)
55	55	404	Mouse	*eEF2-TS2*	44.4% (4/9) - 66.7% (4/6)	-	(Miura et al., 2015)
60	60	779	Mouse	*Mmp13*	-	40% (4/10)	(Quadros et al., 2017)
60	60	782	Mouse	*Mmp9*	-	66.7% (8/12)	(Quadros et al., 2017)
96	98	1220	Mouse	*Otoa*	-	50% (1/2)	(Quadros et al., 2017)
105	98	1368	Mouse	*Fgf8*	-	25% (1/4)	(Quadros et al., 2017)
99	72	1368	Mouse	*Slc26a5*	-	33.3% (1/3)	(Quadros et al., 2017)
85	96	1368	Mouse	*Mafb*	-	25% (2/8)	(Quadros et al., 2017)
203	202	~384-nt Conditional allele	Mouse	*Nomo1*	-	30% (3/10)	(Garcia-Tunon et al., 2020)
55	55	527-nt Conditional allele	Mouse	*Col12a1*	-	100% (3/3)	(Quadros et al., 2017)
78	86	535-nt Conditional allele	Mouse	*Ubr5*	-	100% (2/2)	(Quadros et al., 2017)

75	75	535-nt Conditional allele	Mouse	*Syt1*	-	12.5% (1/8)	(Quadros et al., 2017)
87	68	535-nt Conditional allele	Mouse	*Syt9*	-	8.3% (1/12)	(Quadros et al., 2017)
95	84	535-nt Conditional allele	Mouse	*Ppp2r2a*	-	100% (3/3)	(Quadros et al., 2017)
96	103	589-nt Conditional allele	Mouse	*Ambra1*	-	75% (6/8)	(Quadros et al., 2017)
93	91	862-nt Conditional allele	Mouse	*Pitx1*	-	12.5% (1/8) – 40% (4/10)	(Quadros et al., 2017)
109	113	677-nt Conditional allele	Mouse	*Rapgef5*	-	3.3% (1/30)	(Codner et al., 2018)
108	108	698-nt Conditional allele	Mouse	*Usp45*	-	4% (1/25)	(Codner et al., 2018)
128	129	778-nt Conditional allele	Mouse	*Cx3cl1*	-	15.8% (3/19)	(Codner et al., 2018)
136	108	863-nt Conditional allele	Mouse	*Lkzf2*	-	4.3% (1/23)	(Codner et al., 2018)
119	128	942-nt Conditional allele	Mouse	*6430573F11Rik*	-	3.2% (2/63)	(Codner et al., 2018)
100	100	949-nt Conditional allele	Mouse	*Syt7*	-	11.8% (2/17)	(Codner et al., 2018)

128	129	1137-nt Conditional allele	Mouse	*Inpp5k*	-	8% (2/25)	(Codner et al., 2018)
108	108	1351-nt Conditional allele	Mouse	*Syt4*	-	1.6% (1/66)	(Codner et al., 2018)
128	129	1632-nt Conditional allele	Mouse	*Acvr2b*	-	0% (0/11)	(Codner et al., 2018)
300	60	310-nt Conditional allele	Mouse	*Serpina3n*	-	50% (3/6)	(Miyasaka et al., 2018)
300	60	348-nt Conditional allele	Mouse	*Serpina3n*	-	16.1% (9/56)	(Miyasaka et al., 2018)
300	60	532-nt Conditional allele	Mouse	*Tyr*	-	17.6% (3/17)	(Miyasaka et al., 2018)
300	60	1069-nt Conditional allele	Mouse	*Kiaa1322*	-	80% (4/5)	(Miyasaka et al., 2018)
78	77	480-nt Conditional allele	Mouse	*Vezf1*	-	8.3% (1/12)	(Gurumurthy et al., 2019)
307	60	482-nt Conditional allele	Mouse	*Mettl14*	-	12.3% (8/65)	(Gurumurthy et al., 2019)
329	89	506-nt Conditional allele	Mouse	*Mettl3*	-	9.8% (6/61)	(Gurumurthy et al., 2019)
307	56	599-nt Conditional allele	Mouse	*Tmem163*	-	2.7% (1/37)	(Gurumurthy et al., 2019)

328	77	617-nt Conditional allele	Mouse	*Trmt6*	-	20% (8/40)	(Gurumurthy et al., 2019)
83	70	629-nt Conditional allele	Mouse	*Gene B*	-	10.5% (2/19)	(Gurumurthy et al., 2019)
75	98	642-nt Conditional allele	Mouse	*Bin1*	-	18.2% (2/11)	(Gurumurthy et al., 2019)
314	79	809-nt Conditional allele	Mouse	*Wtap*	-	43.75% (7/16)	(Gurumurthy et al., 2019)
86	88	957-nt Conditional allele	Mouse	*Taok3*	-	13.2% (5/38)	(Gurumurthy et al., 2019)
60	60	717	Rat	*Thy1*	-	0% (0/48)	(Yoshimi et al., 2016)
300	60	834	Rat	*Thy1*	-	11.1% (4/36)	(Yoshimi et al., 2016)

Conditional allele, allele floxed by two LoxP sites.

Table S3 HBR by CRISPR-Cas nuclease and AAV donor in mammalian zygotes

Donor DNA	5' homology arm (nt)	3' homology arm (nt)	Insertion (nt)	Species	Target gene	Efficiency in embryo (Edited/total embryos)	Efficiency in pups (Edited/total pups)	Reference
AAV6	~800	~800	1-nt point mutation	Mouse	*Tyr*	40% (6/15)	5% (1/20)	(Yoon et al., 2018)
AAV6	~800	~800	771	Mouse	*Tyr*	57.1% (4/7)	8.3% (2/24)	(Yoon et al., 2018)
AAV1	480	480	6	Mouse	*Tyr*	77%	-	(Chen et

						(10/13)		al., 2019)
AAV1	477	475	774	Mouse	*Sox2*	33% (6/18)	18% (2/11)	(Chen et al., 2019)
AAV1	477	475	2112	Mouse	*Sox2*	69% (22/32)	40% (2/5)	(Chen et al., 2019)
AAV1	529	500	3300	Mouse	*Rosa26*	34% (9/26)	27% (3/11)	(Chen et al., 2019)

AAV6, AAV serotype 6. AAV1, AAV serotype 1.

Table S4 HBR by CRISPR-Cas nuclease and dsDNA donor in mammalian zygotes

Donor DNA	5' homology arm (bp)	3' homology arm (bp)	Insertion (bp)	Cell type	Target gene	Efficiency in embryo (Edited/total embryos)	Efficiency in pups (Edited/total pups)	Reference
Supercoiled plasmid[a]	60	60	945	Mouse	*Nle*	0% (0/47)	-	(Raveux et al., 2017)
Supercoiled plasmid[a]	250	250	945	Mouse	*Nle*	0% (0/65)	-	(Raveux et al., 2017)
Supercoiled plasmid[a]	500	500	945	Mouse	*Nle*	11.5% (6/52)	-	(Raveux et al., 2017)
Supercoiled plasmid[a]	800	800	~800	Mouse	*Actb*	3.3% (8/239)	-	(Yao et al., 2017)
Supercoiled plasmid[a]	800	800	~800	Mouse	*Dbh*	-	2.27% (1/44)	(Yao et al., 2017)
Supercoiled plasmid[a]	800	800	~800	Mouse	*Sox2*	-	3.33% (1/30)	(Yao et al., 2017)
Supercoiled plasmid[a]	800	800	800	Mouse	*Actb*	0% (0/73)	-	(Yao et al., 2018b)
Supercoiled plasmid[a]	500	500	~2000	Mouse	*Pax9*	-	4.2% (1/24)	(Feng et al., 2016)
Supercoiled	4500	2000	3000	Mouse	*Oct4*	18.5%	30% (3/10)	(Yang et al.,

plasmid[a]						(47/254)		2013)
Supercoiled plasmid[a]	2043	3919	3929	Mouse	*Trp53*	-	40% (2/5)	(Gurumurthy et al., 2019)
Supercoiled plasmid[a]	1800	3100	3000	Mouse	*Rosa26-T8*	-	4.5% (1/22)	(Nakao et al., 2016)
Supercoiled plasmid[a]	8000	3600	3100	Mouse	*Rosa26-T4*	-	2.6% (1/39)	(Nakao et al., 2016)
Supercoiled plasmid[a]	8000	3600	6600	Mouse	*Rosa26-T8*	-	4.8% (1/21)	(Nakao et al., 2016)
Supercoiled plasmid[a]	8100	3600	7100	Mouse	*Rosa26-T2*	-	3.1% (3/97)	(Nakao et al., 2016)
Linear plasmid[a]	1034	4288	7100	Mouse	*Rosa26*	16.7% (1/6)	-	(Menoret et al., 2015)
Linear plasmid[a]	1900	1900	608-bp cond itional allele	Mouse	*lspd*	-	7.7% (1/13)	(Lee and Lloyd, 2014)
Supercoiled plasmid[b]	800	800	~800	Mouse	*Actb*	22.7% (75/331)	-	(Yao et al., 2017)
Supercoiled plasmid[b]	800	800	~700	Mouse	*Dbh*	-	12.1% (4/33)	(Yao et al., 2017)
Supercoiled plasmid[b]	800	800	~800	Mouse	*Sox2*	-	26.9% (7/26)	(Yao et al., 2017)
Supercoiled plasmid[b]	800	800	800	Mouse	*Cdx2*	-	7/43 (16.3%)	(Yao et al., 2018b)
Supercoiled plasmid[b]	800	800	800	Mouse	*Actb*	20.5% (25/122)	-	(Yao et al., 2018b)
Supercoiled plasmid[b]	800	800	~800	Mouse	*Sp8*	-	12.1% (4/33)	(Yao et al., 2018b)
Linear dsDNA[c]	800	800	800	Mouse	*Actb*	33.2% (73/220)	-	(Yao et al., 2018b)
Linear dsDNA[c]	800	800	800	Mouse	*Cdx2*	-	54.4% (31/57)	(Yao et al., 2018b)
Linear dsDNA[c]	800	800	1000	Mouse	*Lhx6*	-	33.3% (4/12)	(Yao et al., 2018b)

Linear dsDNA[c]	800	800	1400	Mouse	*Dbh*	-	20.7% (6/29)	(Yao et al., 2018b)
Linear dsDNA[c]	800	800	2300	Mouse	*Sp8*	-	28.6% (10/35)	(Yao et al., 2018b)
Linear dsDNA[c]	800	800	6000	Mouse	*Rosa26*	-	6.9% (2/29)	(Yao et al., 2018b)
Supercoiled plasmid[d]	20	20	~800	Mouse	*Actb*	11.9% (40/337)	-	(Yao et al., 2017)
Supercoiled plasmid[d]	20	20	~800	Mouse	*Dbh*	-	3.28% (2/61)	(Yao et al., 2017)
Supercoiled plasmid[d]	20	20	~800	Mouse	*Sox2*	-	7.9% (3/38)	(Yao et al., 2017)
Supercoiled plasmid[d]	40	40	5000	Mouse	*Actb*	-	12% (3/25)	(Aida et al., 2016)
Supercoiled plasmid[d,*]	40	40	5000	Mouse	*Actb*	-	35.7% (5/14)	(Aida et al., 2016)
Supercoiled plasmid[d, *]	40	40	520-bp cond itional allele	Mouse	*Col12a1*	-	33.3% (3/9)	(Aida et al., 2016)
Supercoiled plasmid[a]	2143	2143	~700	Rat	*Trdmt1*	-	23.1% (9/39)	(Ma et al., 2014a)
Supercoiled plasmid[a]	1645	1584	~1000	Rat	*Cck*	-	54% (6/11)	(Ma et al., 2014a)
Supercoiled plasmid[a]	1485	1520	~1000	Rat	*Nestin*	-	35% (14/40)	(Ma et al., 2014a)
Supercoiled plasmid[a]	515	645	4800	Rat	*Rosa26*	-	0% (0/25)	(Yoshimi et al., 2016)
Supercoiled plasmid[a]	1000	1000	5300	Rat	*Rosa26*	-	42.9% (15/35)	(Ma et al., 2017b)
Linear plasmid[a]	1000	1000	783	Rat	*FoxP3*	3.5% (1/29)	-	(Menoret et al., 2015)
Linear plasmid[a]	800	800	3142	Rat	*Rosa 26*	3.5% (2/57)	-	(Menoret et al., 2015)
Linear	813	783	3108	Rat	*Rosa26*	-	3.5% (2/57)	(Remy et al.,

plasmid[a]								2017)
Supercoiled plasmid[a]	800	800	~346-bp conditional allele	Rat	*Dnmt1*	-	16.7% (2/12)	(Ma et al., 2014b)
Supercoiled plasmid[a]	783	794	~631-bp conditional allele	Rat	*Dnmt3a*	-	30% (6/20)	(Ma et al., 2014b)
Supercoiled plasmid[a]	800	800	~349-bp conditional allele	Rat	*Dnmt3b*	-	30% (9/30)	(Ma et al., 2014b)
Supercoiled plasmid[a]	2400	1600	~500	Rabbit	*Cftr*	12.5% (5/40)	-	(Song et al., 2016)
Supercoiled plasmid[a]	500	700	~2000	Rabbit	*Rll*	7.1% (2/28)	6.9% (3/43)	(Song et al., 2016)
Supercoiled plasmid[a]	1025	1037	1293	Rabbit	*Csn2*	-	1/15 (6.7%)	(Li et al., 2019)
Supercoiled plasmid[a]	1000	1000	2368	Pig	*Alb*	-	100% (16/16)	(Peng et al., 2015)
Supercoiled plasmid[a]	700	500	520-bp cond itional allele	Pig	*Prnp*	-	25% (1/4)	(Park et al., 2017)
Supercoiled plasmid[b]	800	800	~1000	Monkey	*Actb*	66.7% (24/36)	-	(Yao et al., 2017)
Supercoiled plasmid[b]	800	800	~1500	Monkey	*Actb*	83.3% (5/6)	100% (5/5)	(Yao et al., 2018a)
Supercoiled plasmid[a]	497	498	32-nt deletio n	Human	*CCR5*	6.7% (1/15)	-	(Kang et al., 2016)
Supercoiled plasmid[a]	800	800	~1500	Human	*OCT4*	11.1% (1/9)	-	(Yao et al., 2018b)
Linear dsDNA[c]	800	800	~1500	Human	*OCT4*	71.4% (10/14)	-	(Yao et al., 2018b)

a, HR. b, HMEJ. c, Tild. d, PITCh. *, Coinjected with Exo1 mRNA.

REFERENCES

Aida, T., Nakade, S., Sakuma, T., Izu, Y., Oishi, A., Mochida, K., Ishikubo, H., Usami, T., Aizawa, H., Yamamoto, T., *et al.* (2016). Gene cassette knock-in in mammalian cells and zygotes by enhanced MMEJ. BMC Genomics *17*, 979.

Chen, S., Sun, S., Moonen, D., Lee, C., Lee, A.Y., Schaffer, D.V., and He, L. (2019). CRISPR-READI: Efficient Generation of Knockin Mice by CRISPR RNP Electroporation and AAV Donor Infection. Cell Rep *27*, 3780-3789 e3784.

Codner, G.F., Mianne, J., Caulder, A., Loeffler, J., Fell, R., King, R., Allan, A.J., Mackenzie, M., Pike, F.J., McCabe, C.V., *et al.* (2018). Application of long single-stranded DNA donors in genome editing: generation and validation of mouse mutants. BMC Biol *16*, 70.

Feng, J., Jing, J., Sanchez-Lara, P.A., Bootwalla, M.S., Buckley, J., Wu, N., Yan, Y., and Chai, Y. (2016). Generation and characterization of tamoxifen-inducible Pax9-CreER knock-in mice using CrispR/Cas9. Genesis *54*, 490-496.

Garcia-Tunon, I., Vuelta, E., Lozano, L., Herrero, M., Mendez, L., Palomero-Hernandez, J., Perez-Caro, M., Perez-Garcia, J., Gonzalez-Sarmiento, R., and Sanchez-Martin, M. (2020). Establishment of a conditional Nomo1 mouse model by CRISPR/Cas9 technology. Mol Biol Rep *47*, 1381-1391.

Ge, X.A., and Hunter, C.P. (2019). Efficient Homologous Recombination in Mice Using Long Single Stranded DNA and CRISPR Cas9 Nickase. G3 (Bethesda) *9*, 281-286.

Gurumurthy, C.B., O'Brien, A.R., Quadros, R.M., Adams, J., Jr., Alcaide, P., Ayabe, S., Ballard, J., Batra, S.K., Beauchamp, M.C., Becker, K.A., *et al.* (2019). Reproducibility of CRISPR-Cas9 methods for generation of conditional mouse alleles: a multi-center evaluation. Genome Biol *20*, 171.

Han, Y., Slivano, O.J., Christie, C.K., Cheng, A.W., and Miano, J.M. (2015). CRISPR-Cas9 genome editing of a single regulatory element nearly abolishes target gene expression in mice--brief report. Arterioscler Thromb Vasc Biol *35*, 312-315.

Inui, M., Miyado, M., Igarashi, M., Tamano, M., Kubo, A., Yamashita, S., Asahara, H., Fukami, M., and Takada, S. (2014). Rapid generation of mouse models with defined point mutations by the CRISPR/Cas9 system. Sci Rep *4*, 5396.

Kang, X., He, W., Huang, Y., Yu, Q., Chen, Y., Gao, X., Sun, X., and Fan, Y. (2016). Introducing precise genetic modifications into human 3PN embryos by CRISPR/Cas-mediated genome editing. J Assist Reprod Genet *33*, 581-588.

Lamas-Toranzo, I., Martinez-Moro, A., E, O.C., Millan-Blanca, G., Sanchez, J.M., Lonergan, P., and Bermejo-Alvarez, P. (2020). RS-1 enhances CRISPR-mediated targeted knock-in in bovine embryos. Mol Reprod Dev *87*, 542-549.

Lee, A.Y., and Lloyd, K.C. (2014). Conditional targeting of Ispd using paired Cas9 nickase and a single DNA template in mice. FEBS Open Bio *4*, 637-642.

Li, F., Cowley, D.O., Banner, D., Holle, E., Zhang, L., and Su, L. (2014). Efficient genetic manipulation of the NOD-Rag1-/-IL2RgammaC-null mouse by combining in vitro fertilization and CRISPR/Cas9 technology. Sci Rep *4*, 5290.

Li, H., Li, Z., Xiao, N., Su, X., Zhao, S., Zhang, Y., Cui, K., Liu, Q., and Shi, D. (2019). Site-specific integration of rotavirus VP6 gene in rabbit beta-casein locus by CRISPR/Cas9 system. In vitro cellular & developmental biology Animal *55*, 586-597.

Liang, P., Xu, Y., Zhang, X., Ding, C., Huang, R., Zhang, Z., Lv, J., Xie, X., Chen, Y., Li, Y., *et al.* (2015). CRISPR/Cas9-mediated gene editing in human tripronuclear zygotes. Protein Cell *6*, 363-372.

Long, C., McAnally, J.R., Shelton, J.M., Mireault, A.A., Bassel-Duby, R., and Olson, E.N. (2014). Prevention of muscular dystrophy in mice by CRISPR/Cas9-mediated editing of germline DNA. Science *345*, 1184-1188.

Ma, X., Chen, C., Veevers, J., Zhou, X., Ross, R.S., Feng, W., and Chen, J. (2017a). CRISPR/Cas9-mediated gene manipulation to create single-amino-acid-substituted and floxed mice with a cloning-free method. Sci Rep *7*, 42244.

Ma, Y., Ma, J., Zhang, X., Chen, W., Yu, L., Lu, Y., Bai, L., Shen, B., Huang, X., and Zhang, L. (2014a). Generation of eGFP and Cre knockin rats by CRISPR/Cas9. The FEBS journal *281*, 3779-3790.

Ma, Y., Yu, L., Pan, S., Gao, S., Chen, W., Zhang, X., Dong, W., Li, J., Zhou, R., Huang, L., *et al.* (2017b). CRISPR/Cas9-mediated targeting of the Rosa26 locus produces Cre reporter rat strains for monitoring Cre-loxP-mediated lineage tracing. The FEBS journal *284*, 3262-3277.

Ma, Y., Zhang, X., Shen, B., Lu, Y., Chen, W., Ma, J., Bai, L., Huang, X., and Zhang, L. (2014b). Generating rats with conditional alleles using CRISPR/Cas9. Cell research *24*, 122-125.

Menchaca, A., Dos Santos-Neto, P.C., Souza-Neves, M., Cuadro, F., Mulet, A.P., Tesson, L., Chenouard, V., Guiffes, A., Heslan, J.M., Gantier, M., *et al.* (2020). Otoferlin gene editing in sheep via CRISPR-assisted ssODN-mediated Homology Directed Repair. Sci Rep *10*, 5995.

Menoret, S., De Cian, A., Tesson, L., Remy, S., Usal, C., Boule, J.B., Boix, C., Fontaniere, S., Creneguy, A., Nguyen, T.H., *et al.* (2015). Homology-directed repair in rodent zygotes using Cas9 and TALEN engineered proteins. Sci Rep *5*, 14410.

Miao, K., Zhang, X., Su, S.M., Zeng, J., Huang, Z., Chan, U.I., Xu, X., and Deng, C.X. (2019). Optimizing CRISPR/Cas9 technology for precise correction of the Fgfr3-G374R mutation in achondroplasia in mice. J Biol Chem *294*, 1142-1151.

Miura, H., Gurumurthy, C.B., Sato, T., Sato, M., and Ohtsuka, M. (2015). CRISPR/Cas9-based generation of knockdown mice by intronic insertion of artificial microRNA using longer single-stranded DNA. Sci Rep *5*, 12799.

Miyasaka, Y., Uno, Y., Yoshimi, K., Kunihiro, Y., Yoshimura, T., Tanaka, T., Ishikubo, H., Hiraoka, Y., Takemoto, N., Tanaka, T., *et al.* (2018). CLICK: one-step generation of conditional knockout mice. BMC Genomics *19*, 318.

Nakagawa, Y., Sakuma, T., Nishimichi, N., Yokosaki, Y., Yanaka, N., Takeo, T., Nakagata, N., and Yamamoto, T. (2016). Ultra-superovulation for the CRISPR-Cas9-mediated production of gene-knockout, single-amino-acid-substituted, and floxed mice. Biol Open *5*, 1142-1148.

Nakao, H., Harada, T., Nakao, K., Kiyonari, H., Inoue, K., Furuta, Y., and Aiba, A. (2016). A possible aid in targeted insertion of large DNA elements by CRISPR/Cas in mouse zygotes. Genesis *54*, 65-77.

Park, K.E., Powell, A., Sandmaier, S.E., Kim, C.M., Mileham, A., Donovan, D.M., and Telugu, B.P. (2017). Targeted gene knock-in by CRISPR/Cas ribonucleoproteins in porcine zygotes. Sci Rep *7*, 42458.

Peng, J., Wang, Y., Jiang, J., Zhou, X., Song, L., Wang, L., Ding, C., Qin, J., Liu, L., Wang, W., *et al.* (2015). Production of Human Albumin in Pigs Through CRISPR/Cas9-Mediated Knockin of Human cDNA into Swine Albumin Locus in the Zygotes. Sci Rep *5*, 16705.

Pritchard, C.E.J., Kroese, L.J., and Huijbers, I.J. (2017). Direct Generation of Conditional Alleles Using CRISPR/Cas9 in Mouse Zygotes. Methods Mol Biol *1642*, 21-35.

Quadros, R.M., Harms, D.W., Ohtsuka, M., and Gurumurthy, C.B. (2015). Insertion of sequences at the original provirus integration site of mouse ROSA26 locus using the CRISPR/Cas9 system. FEBS Open Bio *5*, 191-197.

Quadros, R.M., Miura, H., Harms, D.W., Akatsuka, H., Sato, T., Aida, T., Redder, R., Richardson, G.P., Inagaki, Y., Sakai, D., *et al.* (2017). Easi-CRISPR: a robust method for one-step generation of mice carrying conditional and insertion alleles using long ssDNA donors and CRISPR ribonucleoproteins. Genome Biol *18*, 92.

Raveux, A., Vandormael-Pournin, S., and Cohen-Tannoudji, M. (2017). Optimization of the production of knock-in alleles by CRISPR/Cas9 microinjection into the mouse zygote. Sci Rep *7*, 42661.

Remy, S., Chenouard, V., Tesson, L., Usal, C., Menoret, S., Brusselle, L., Heslan, J.M., Nguyen, T.H., Bellien, J., Merot, J., *et al.* (2017). Generation of gene-edited rats by delivery of CRISPR/Cas9 protein and donor DNA into intact zygotes using electroporation. Sci Rep *7*, 16554.

Renaud, J.B., Boix, C., Charpentier, M., De Cian, A., Cochennec, J., Duvernois-Berthet, E., Perrouault, L., Tesson, L., Edouard, J., Thinard, R., *et al.* (2016). Improved Genome Editing Efficiency and Flexibility Using Modified Oligonucleotides with TALEN and CRISPR-Cas9 Nucleases. Cell Rep *14*, 2263-2272.

Shao, Y., Guan, Y., Wang, L., Qiu, Z., Liu, M., Chen, Y., Wu, L., Li, Y., Ma, X., Liu, M., *et al.* (2014). CRISPR/Cas-mediated genome editing in the rat via direct injection of one-cell embryos. Nat Protoc *9*, 2493-2512.

Singh, P., and Schimenti, J.C. (2015). The genetics of human infertility by functional interrogation of SNPs in mice. Proc Natl Acad Sci U S A *112*, 10431-10436.

Song, J., Yang, D., Xu, J., Zhu, T., Chen, Y.E., and Zhang, J. (2016). RS-1 enhances CRISPR/Cas9- and TALEN-mediated knock-in efficiency. Nat Commun *7*, 10548.

Song, Y., Zhang, Y., Chen, M., Deng, J., Sui, T., Lai, L., and Li, Z. (2018). Functional validation of the albinism-associated tyrosinase T373K SNP by CRISPR/Cas9-mediated homology-directed repair (HBR) in rabbits. EBioMedicine *36*, 517-525.

Tang, L., Zeng, Y., Du, H., Gong, M., Peng, J., Zhang, B., Lei, M., Zhao, F., Wang, W., Li, X., *et al.* (2017). CRISPR/Cas9-mediated gene editing in human zygotes using Cas9 protein. Mol Genet Genomics *292*, 525-533.

Wang, H., Yang, H., Shivalila, C.S., Dawlaty, M.M., Cheng, A.W., Zhang, F., and Jaenisch, R. (2013). One-step generation of mice carrying mutations in multiple genes by CRISPR/Cas-mediated genome engineering. Cell *153*, 910-918.

Wu, Y., Liang, D., Wang, Y., Bai, M., Tang, W., Bao, S., Yan, Z., Li, D., and Li, J. (2013). Correction of a genetic disease in mouse via use of CRISPR-Cas9. Cell Stem Cell *13*, 659-662.

Yang, H., Wang, H., Shivalila, C.S., Cheng, A.W., Shi, L., and Jaenisch, R. (2013). One-step generation of mice carrying reporter and conditional alleles by CRISPR/Cas-mediated genome engineering. Cell *154*, 1370-1379.

Yao, X., Liu, Z., Wang, X., Wang, Y., Nie, Y.H., Lai, L., Sun, R., Shi, L., Sun, Q., and Yang, H. (2018a). Generation of knock-in cynomolgus monkey via CRISPR/Cas9 editing. Cell research *28*, 379-382.

Yao, X., Wang, X., Hu, X., Liu, Z., Liu, J., Zhou, H., Shen, X., Wei, Y., Huang, Z., Ying, W., *et al.* (2017). Homology-mediated end joining-based targeted integration using CRISPR/Cas9. Cell Res *27*, 801-814.

Yao, X., Zhang, M., Wang, X., Ying, W., Hu, X., Dai, P., Meng, F., Shi, L., Sun, Y., Yao, N., *et al.* (2018b). Tild-CRISPR Allows for Efficient and Precise Gene Knockin in Mouse and Human Cells. Dev Cell *45*, 526-536 e525.

Yoon, Y., Wang, D., Tai, P.W.L., Riley, J., Gao, G., and Rivera-Perez, J.A. (2018). Streamlined ex vivo and in vivo genome editing in mouse embryos using recombinant adeno-associated viruses. Nat Commun *9*, 412.

Yoshimi, K., Kaneko, T., Voigt, B., and Mashimo, T. (2014). Allele-specific genome editing and correction of disease-associated phenotypes in rats using the CRISPR-Cas platform. Nat Commun *5*, 4240.

Yoshimi, K., Kunihiro, Y., Kaneko, T., Nagahora, H., Voigt, B., and Mashimo, T. (2016). ssODN-mediated knock-in with CRISPR-Cas for large genomic regions in zygotes. Nat Commun *7*, 10431.

Zhang, X., Liang, P., Ding, C., Zhang, Z., Zhou, J., Xie, X., Huang, R., Sun, Y., Sun, H., Zhang, J., *et al.* (2016). Efficient Production of Gene-Modified Mice using Staphylococcus aureus Cas9. Sci Rep *6*, 32565.

Zhou, X., Wang, L., Du, Y., Xie, F., Li, L., Liu, Y., Liu, C., Wang, S., Zhang, S., Huang, X., *et al.* (2016). Efficient Generation of Gene-Modified Pigs Harboring

Precise Orthologous Human Mutation via CRISPR/Cas9-Induced Homology-Directed Repair in Zygotes. Hum Mutat *37*, 110-118.
